# Tracking Clonal Evolution of Multiple Myeloma Using Targeted Next-Generation DNA Sequencing

**DOI:** 10.3390/biomedicines10071674

**Published:** 2022-07-12

**Authors:** Aleksander Salomon-Perzyński, Joanna Barankiewicz, Marcin Machnicki, Irena Misiewicz-Krzemińska, Michał Pawlak, Sylwia Radomska, Agnieszka Krzywdzińska, Aleksandra Bluszcz, Piotr Stawiński, Małgorzata Rydzanicz, Natalia Jakacka, Iwona Solarska, Katarzyna Borg, Zofia Spyra-Górny, Tomasz Szpila, Bartosz Puła, Sebastian Grosicki, Tomasz Stokłosa, Rafał Płoski, Ewa Lech-Marańda, Jana Jakubikova, Krzysztof Jamroziak

**Affiliations:** 1 Department of Hematology, Institute of Hematology and Transfusion Medicine, 02-776 Warsaw, Poland; asalomon@ihit.waw.pl (A.S.-P.); jbarankiewicz@ihit.waw.pl (J.B.); njakacka@ihit.waw.pl (N.J.); tszpila@ihit.waw.pl (T.S.); bpula@ihit.waw.pl (B.P.); emaranda@ihit.waw.pl (E.L.-M.); 2 Department of Tumor Biology and Genetics, Medical University of Warsaw, 02-106 Warsaw, Poland; marmach.marmach@gmail.com (M.M.); tomasz.stoklosa@wum.edu.pl (T.S.); 3 Department of Experimental Hematology, Institute of Hematology and Transfusion Medicine, 02-776 Warsaw, Poland; imisiewiczk@ihit.waw.pl (I.M.-K.); mpawlak@get2omics.com (M.P.); 4 Molecular Biology Laboratory, Department of Diagnostic Hematology, Institute of Hematology and Transfusion Medicine, 02-776 Warsaw, Poland; sradomska@ihit.waw.pl (S.R.); isolarska@hotmail.com (I.S.); 5 Immunophenotyping Laboratory, Department of Diagnostic Hematology, Institute of Hematology and Transfusion Medicine, 02-776 Warsaw, Poland; akrzywdzinska@ihit.waw.pl; 6 Cytogenetic Laboratory, Department of Diagnostic Hematology, Institute of Hematology and Transfusion Medicine, 02-776 Warsaw, Poland; a.bluszcz@yahoo.pl (A.B.); kborg@ihit.waw.pl (K.B.); 7 Department of Medical Genetics, Medical University of Warsaw, 02-106 Warsaw, Poland; stawinski84@gmail.com (P.S.); mrydzanicz@wum.edu.pl (M.R.); rploski@wp.pl (R.P.); 8 Department of Hematology and Cancer Prevention, Faculty od Health Sciences, Medical University of Silesia in Katowice, 40-055 Katowice, Poland; zspyra@gmail.com (Z.S.-G.); sgrosicki@wp.pl (S.G.); 9 Department of Tumor Immunology, Biomedical Research Center, Cancer Research Institute, Slovak Academy of Sciences, Dubravska Cesta 9, 84505 Bratislava, Slovakia; jana.jakubikova@savba.sk; 10 Department of Hematology, Transplantation and Internal Medicine, Medical University of Warsaw, 02-106 Warsaw, Poland

**Keywords:** clonal evolution, multiple myeloma, next-generation sequencing

## Abstract

Clonal evolution drives treatment failure in multiple myeloma (MM). Here, we used a custom 372-gene panel to track genetic changes occurring during MM progression at different stages of the disease. A tumor-only targeted next-generation DNA sequencing was performed on 69 samples sequentially collected from 30 MM patients. The MAPK/ERK pathway was mostly affected with KRAS mutated in 47% of patients. Acquisition and loss of mutations were observed in 63% and 37% of patients, respectively. Four different patterns of mutation evolution were found: branching-, mutation acquisition-, mutation loss- and a stable mutational pathway. Better response to anti-myeloma therapy was more frequently observed in patients who followed the mutation loss—compared to the mutation acquisition pathway. More than two-thirds of patients had druggable genes mutated (including cases of heavily pre-treated disease). Only 7% of patients had a stable copy number variants profile. Consequently, a redistribution in stages according to R-ISS between the first and paired samples (R-ISS″) was seen. The higher the R-ISS″, the higher the risk of MM progression and death. We provided new insights into the genetics of MM evolution, especially in heavily pre-treated patients. Additionally, we confirmed that redefining R-ISS at MM relapse is of high clinical value.

## 1. Introduction

In the era of expanding therapeutic armamentarium, the prognosis of patients with multiple myeloma (MM) continues to improve, but the emergence of acquired drug resistance inevitably complicates the clinical course of MM in almost every case [1]. As demonstrated by next-generation DNA sequencing (NGS), MM is a spatially heterogeneous composite of genetically distinct subclones (i.e., subpopulations of MM cells harboring the same set of genomic variants, including single-nucleotide variants (SNV), structural variants and copy number variants (CNV)) evolving over time following different patterns of clonal evolution with a branching pattern being the most common [2,3,4,5,6,7,8,9,10]. Competition between subclones for limited sources of the local microenvironment is the main driving force of clonal evolution. Generally, those of the subclones that are phenotypically best adapted to local microenvironmental conditions are positively selected and undergo further expansion, eliminating less fit subclones from the population of MM cells [11]. However, as cancers act as microecosystems, it is expected that the dynamics of MM evolution are also influenced by more complex factors such as cooperation between subclones themselves and between subclones and the various components of the local microenvironment [12]. During therapy, the molecular architecture of MM changes due to the direct elimination of susceptible subclones but also due to disruption of a wide network of subclone-subclone and subclone-microenvironment interactions. Treatment failure is thought to occur through a selection of drug-resistant subclones initially present in the tumor mass and/or through arising of drug-resistant subclones de novo during anti-myeloma therapy [3,4,8,9,13,14,15,16,17]. Therefore, since the emergence of drug resistance is driven by evolutionary dynamics, tracking changes in the subclonal architecture of MM at different time points during treatment (e.g., at measurable residual disease and at biochemical and clinical relapse) and during the follow-up is of great value. Recently, several NGS studies provided insight into the spectrum of genomic changes between MM diagnosis and relapse [3,4,8,9,13,14,15,16,17]. However, no unified rules have yet been identified to explain how the acquisition, persistence or loss of certain genetic events during clonal evolution underlies resistance to anti-myeloma agents. Studies vary in terms of their methodology, the type of used NGS technology (e.g., whole-exome sequencing (WES) and targeted sequencing) and the clinical characteristics of included patients. In addition, MM is characterized by substantial inter-patient genetic heterogeneity [2,6,9,14,18], and as a result, specific repertoire of genetic events is rarely repeated between patients. The fact that patients undergo various therapeutic approaches further complicates the direct comparison of NGS studies in MM. Therefore, much more NGS data from longitudinally collected samples are required to decipher the genomic basis of arising resistance to anti-myeloma therapy. Accordingly, we performed a tumor-only targeted sequencing on sequentially collected myeloma cells to provide insight into the spectrum of genetic changes that occur in MM patients at different stages of the disease.

## 2. Materials and Methods

We selected patients with MM for whom CD138(+) plasma cells from bone marrow samples were available at least at two different time points. Part of the samples was collected prospectively (as part of the ERA-NET grant TRANSCAN2/intraMMclo/2/2017), while the others were derived from the archives of the Institute of Hematology and Transfusion Medicine in Warsaw (Poland). The first sample was obtained at the time of MM diagnosis or relapse, while each subsequent sample was taken at any relapse following the first sample. For patients with more than two samples collected, only a pair of samples (paired samples) was selected for statistical analyses.

Patients’ clinical and laboratory data were collected at the time of the first and paired sample collection using electronic Case Report Forms (CRFs).

Patients’ ECOG (Eastern Cooperative Oncology Group) performance status and MM stage according to the International Staging System (ISS) and Revised ISS (R-ISS) were evaluated as described in the references [19,20,21].

### 2.1. Targeted Sequencing

Plasma cells were magnetically isolated from bone marrow aspirates *with* CD138 microbeads (Human Whole Blood and Bone Marrow CD138 Positive Selection Kit II, Stemcell Technologies, Vancouver, Canada). Post-sorting purity was assessed cytologically. Median purity was 90% (range, 82–98%). DNA was extracted, and libraries were prepared using QIAamp DNA Kits (Qiagen, Hilden, Germany) and KAPA HyperPlus Kit (Roche, Basel, Switzerland), respectively, in accordance with the manufacturer’s recommendations. Germinal DNA was unavailable for analysis. For targeted sequencing, we used a custom gene panel containing coding sequences of a total of 372 genes, including those recurrently mutated in MM [5,7,8,15,22,23,24] and those involved in interactions between MM cells and bone marrow microenvironment [25] (Table S1). Additionally, for copy number analysis, the panel contained 2630 single-nucleotide polymorphisms uniformly distributed throughout the genome. All libraries were sequenced (paired-end 2 × 100 bp) on Illumina NovaSeq 6000. The sequencing data were aligned to the human GRCh38 reference genome using the BWA-MEM aligner (version 0.7.17; https://github.com/lh3/bwa/releases/tag/v0.7.17 (accessed on 10 April 2021)) [26]. Picard (version 2.25.0; Broad Institute, Cambridge, MA, USA; https://broadinstitute.github.io/picard/ (accessed on 10 April 2021)) was used to remove duplicated reads. Raw data (paired FASTQ files per sample) have been deposited on the Institute of Hematology and Transfusion Medicine internal server. Mapped reads were pre-processed for variant calling according to GATK best practice workflows (Broad Institute, Cambridge, MA, USA; https://gatk.broadinstitute.org/hc/en-us/articles/360035535912-Data-pre-processing-for-variant-discovery (accessed on 10 April 2021)). Variant calling was performed in tumor-only mode using MuTect2 (version 4.2.0; Broad Institute, Cambridge, MA, USA; https://gatk.broadinstitute.org/hc/en-us/articles/4409917447707-Mutect2 (accessed on 10 April 2021)). The Ensembl Variant Effect Predictor was used for further variants analysis [27]. Only variants fulfilling the following criteria were included in the analysis: (1) allele frequency in the general population <1% (according to the 1000 Genomes, GnomAD and ESP databases), (2) VAF ≥ 10% in at least one of the paired samples with ≥5 variant reads, (3) pathogenic status in at least 2 predictors among the following: Cancer-Related Analysis of VAriants Toolkit (CRAVAT) [28,29], Cancer-specific High-Throughput Annotation of Somatic Mutations (CHASM) [30,31], CScape [32], FATHMM Cancer [33], DEOGEN2 [34] and PrimateAI [35]. For genes recurrently mutated in MM, variants found to be pathogenic in at least one cancer-specific predictor such as CRAVAT, CHASM, FATHMM Cancer or CScape were allowed for analysis. Variants fulfilling these criteria (*n* = 257) were further verified in the Varsome database [36], and all but one (*KMT2C* c.1173C>A) of those with “benign” or “likely benign” status were rejected (*n* = 78). Copy number variants (CNV) were called using CNVkit (version 0.9.9; https://github.com/etal/cnvkit (accessed on 15 April 2021)) [37]. The identification of potentially actionable targets was based on the Broad Institute’s TARGET (Tumor Alterations Relevant for Genomics driven Therapy) database (https://software.broadinstitute.org/cancer/cga/target (accessed on 10 January 2022)) and OncoKB database (https://www.oncokb.org (accessed on 10 January 2022)) [38]. The functional categorization of mutant genes was based on the WEB-based Gene SeT AnaLysis Toolkit (http://www.webgestalt.org/ (accessed on 3 March 2022)) and Reactome (https://reactome.org (accessed on 3 March 2022)).

### 2.2. Cytogenetic Evaluation

In addition to targeted NGS, samples were also tested by Fluorescence in situ hybridization (FISH) using the following panel of probes: 11q22.3 (*ATM*), 17p13.1 (*TP53*), 14q32 (*IGH*), *FGFR3*/*IGH* t(4;14) and *IGH*/*MAF* t(14;16). According to the local standard, sequential testing strategy was implemented. Thus, t(4;14) and t(14;16) were tested only when *IGH* rearrangement was present in the absence of *TP53* deletion. Some samples which were found to have 14q32 rearrangement other than t(4;14) and t(14;16) were screened for t(11;14). The cut-off values established in the local laboratory for a positive FISH result were 7% for 17p13.1 (*TP53*) deletion, 4% for 11q22.3 (*ATM*) deletion, 8% for any 14q32 (*IGH*) translocations.

### 2.3. Clinical Endpoints

For the purpose of this analysis, we used definitions of treatment endpoints consistent with the 2016 Revised International Myeloma Working Group Criteria [39]. The refractory disease was defined as a lack of any response to treatment or MM progression during treatment or within 60 days after treatment cessation. Progression-free survival (PFS) was defined as the time between initiation of therapy received immediately after the paired sample evaluation and progression or death. Overall survival′ (OS′) was defined as the time from the first sample evaluation to death of any cause. OS″ was defined as the time from the paired sample evaluation to death of any cause.

### 2.4. Statistical Analysis

Categorical variables were compared using the chi-squared or Fisher test depending on the number of observations in each 2-by-2 table. Continued variables were compared using the t-Student test if they followed normal distribution or the Wilcoxon test if they did not follow normal distribution. The distribution of the variables was checked by plotting histograms. Survival function with 95% confidence intervals (95% CI) was estimated using the Kaplan–Meyer method. To estimate hazard ratios (HR) and 95% CI, the proportional hazard Cox model was used. All tests were two-sided and were performed at a 0.05 significance level. All analyzes were performed using software: Statistica v. 13.1 (StatSoft Polska, Kraków, Polska) and MedCalc v. 20.027 (MedCalc Software, Ostend, Belgium). The images were plotted in GraphPad Prism v. 9.3 (GraphPad Software, San Diego, California, USA) and in SmartArt Graphics (Microsoft Word v. 16.60; Microsoft Corporation, Redmond, WA, USA).

## 3. Results

From 4 April 2013 to 11 November 2020, a total of 69 samples from 30 patients were collected. In 19 (63%) patients, the diagnostic sample was paired with at least one progressive sample (a total of 2, 3 and 4 samples were collected for 15, 2 and 2 patients, respectively), while in the remaining 11 patients, only the progressive samples were paired (a total of 2 and 3 samples were collected for 8 and 3 patients, respectively). Patient and disease characteristics are summarized in Table 1. An average read sequencing depth was 267×. Coverage of at least 20× in at least 90% and 95% of sequences was achieved in 69 (100%) and 16 (23%) samples, respectively.

### 3.1. Single-Nucleotide Variants

**Figure 1:**
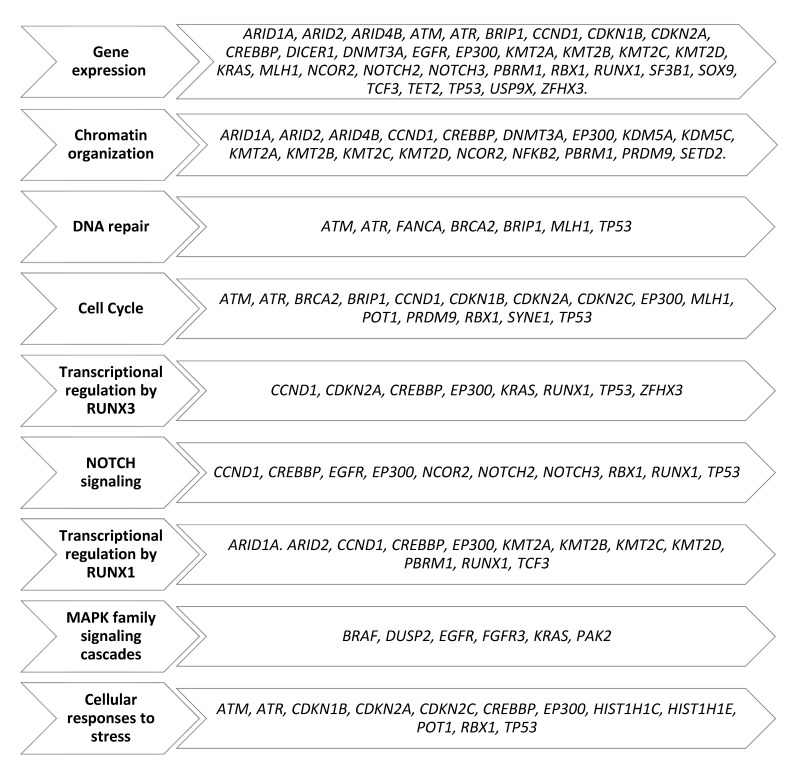
The functional categorization of all mutant genes.

**Figure 2:**
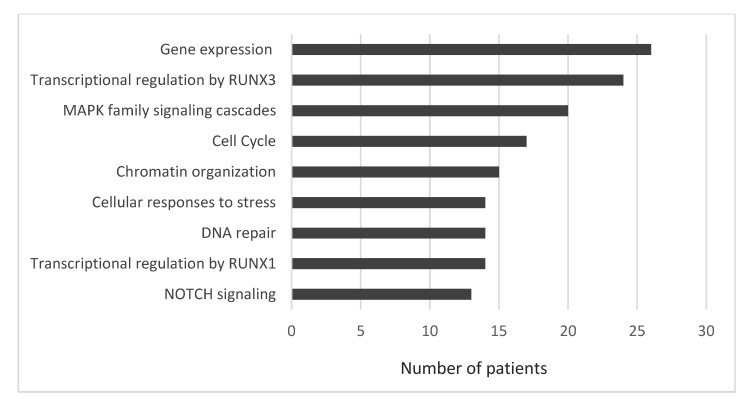
The proportions of multiple myeloma patients bearing mutations in genes involved in a particular cellular process.

A total of 179 different variants of 98 genes were identified. In general, the median number of variants per patient was 5 (range, 2–33). In patients for whom a diagnostic sample was available, a median number of variants in the first, second, third and fourth sample was 4 (range, 1–16), 4 (range, 2–29), 6 (range, 1–9) and 8 (range, 3–12), respectively. In turn, a median number of 6 (range, 2–32), 7 (range, 3–27) and 6 (range, 4–21) variants was found in the first, second and third sample in patients for whom only relapsed samples were available. For all relapsed samples, the median number of variants was 5 (range, 1–32). The functional categorization of identified mutant genes is summarized in Figure 1. The proportions of patients bearing mutations in genes involved in a particular cellular process are summarized in Figure 2.

**Figure 3:**
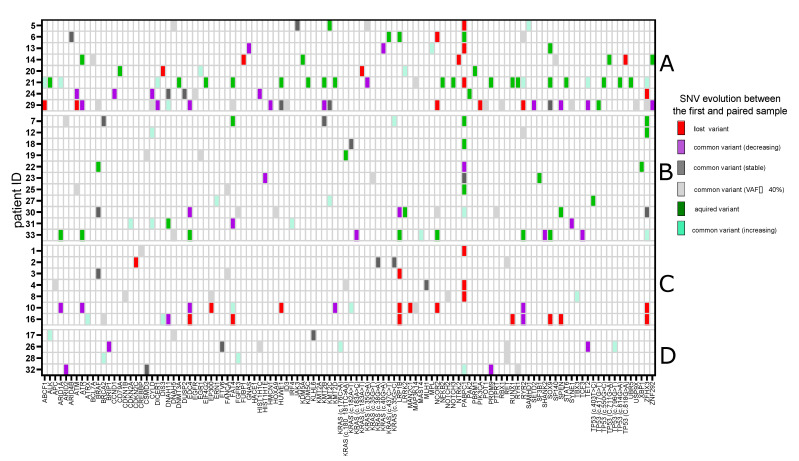
Evolution of single-nucleotide variants during multiple myeloma progression based on the next-generation sequencing data from the first and paired samples. Description: A—patients who presented a branching evolution with mutations lost and gained during multiple myeloma progression; B—patients who only acquired new mutations during multiple myeloma progression; C—patients who only lost mutations during multiple myeloma progression; D—patients with stable mutations during multiple myeloma progression.

We found a total of 141 variants of 78 genes common to paired samples from an individual patient. Thirteen of these genes were mutated in at least 10% of patients, with *KRAS* (*n* = 11, 37%) being the most common, followed by *RYR2* (*n* = 5, 17%), *PABPC1* (*n* = 5, 17%), *ZFHX3* (*n* = 4, 13%), *ATM* (*n* = 3, 10%), *BRCA2* (*n* = 3, 10%), *CYLD* (*n* = 3, 10%), *DNAH5* (*n* = 3, 10%), *DNAH11* (*n* = 3, 10%), *EDC4* (*n* = 3, 10%), *FAT4* (*n* = 3, 10%), *TET2* (*n* = 3, 10%) and *RET* (*n* = 3, 10%). Some of the common variants underwent selection and outgrowth (35/141, 25%) (e.g., *KRAS* c.182A>T (patient 10), c.35G>C (patient 7), c.176C>A (patient 26), *BRCA2* c.8182G>A (patient 28), *KMT2C* c.1014G>A (patient 27), *TET2* c.1841G>A (patient 21), *CYLD* c.1240C>T (patient 31), *TP53* c.796G>T (patient 26)), some declined (41/141, 29%), but most (65/141, 46%), tended to be stable during MM progression (Figure 3). Genes of MAPK signaling pathway (*KRAS*, *BRAF*, *EGFR*, *FGFR3* and *DUSP2*) were most frequently affected (53%, 16/30 patients). Two patients had two genes altered (patient 30: *FGFR3* and *BRAF*; patient 24: *DUSP2* and *EGFR*), while one patient had two mutations in one gene (patient 24: *DUSP2*). DNA repair pathway (*ATM, ATR, FANCA, BRCA2, BRIP1, MLH1* and *TP53*) and epigenetic regulators (*ARID1A,* *ARID2, ARID4B,* *CCND1, CREBBP,* *EP300, KMT2B,* *KMT2C,* *KMT2D, NCOR2* and *PRDM9*) were affected in 10 (33%) and 11 (37%) patients, respectively.

Acquisition of mutations over time was observed in 19 (63%) patients with a median of 1 (range, 1–19) new variants acquired per sample pair (Figure 3). The top eight genes in which mutations were acquired were as follows: *TP53, PABPC1, SOX9* (10% of patients each), *ARID1A, ATR, FAT4, KRAS, LRP1B* (7% of patients each). In 11 patients, at least 1 mutation disappeared over time, with pathogenic variants of *PABPC1* disappearing most frequently (*n* = 5, 17%), followed by *LRP1B* (*n* = 3, 10%), *ZFHX3* (*n* = 3, 10%) and *NCOR2* (*n* = 2, 7%) (Figure 3).

In eight (27%) patients, clear evidence of branching clonal evolution with some subclonal and clonal mutations disappearing and others appearing between paired samples was demonstrated (Figure 3). In addition, signs of parallel evolution were observed with the acquisition of two independent subclonal mutations in *TP53* (patient 21: c.550G>C vs. c.814G>A) and *PABPC1* (patient 18: c.1240C>T vs. c.1223A>T). Parallel evolution was even more pronounced in the samples taken on the later relapses (i.e., *KMT2D* (patient 30: c.9370G>A vs.c.5875G>A), *CYLD* (patient 16: c.2065C>T vs. c.2465A>G)) (Figure S1). Eighteen (60%) patients followed different patterns of progression, namely, the mutation acquisition pathway (*n* = 11, 37%) and the mutation loss pathway (*n* = 7, 23%). Finally, in four (13%), the stable mutational composition pathway occurred as there was no significant change in the subclonal structure of MM at the time of progression. There were, however, changes in the proportion of mutations observed, particularly in patients 26 and 28 (Figure 3).

The evolution of SNV during MM progression in patients with more than two samples collected is shown in Figure S1.

In our cohort, patients who followed the mutation loss pathway more frequently achieved better response (≥very good partial response (VGPR)) to the anti-myeloma therapy received between paired samples compared to patients following the mutation acquisition pathway who achieved at most a PR (*p* = 0.01).

### 3.2. Single-Nucleotide Variants of Druggable Genes

Focusing on point mutations that survived selection imposed by anti-myeloma therapy, 23% (32/141; 20 patients) and 28% (40/141; 21 patients) of them affected druggable genes according to the OnkoKB and TARGET database, respectively (Table S2). Some of these variants decreased (OnkoKB: 8/32; 7 patients; TARGET: 11/40; 6 patients) but most of them underwent selection and expansion (OncoKB: 9/32; 7 patients; TARGET: 9/40; 8 patients) or tended to be stable (OnkoKB: 15/32; 12 patients; TARGET: 20/40, 15 patients) during MM progression. Furthermore, there were clear examples of subclonal mutations (i.e., patient 10: c.182A>T *KRAS;* patient 31: c.98A>G *CDKN2A*) becoming clonal during disease progression (Table S2).

Similarly to the shared mutations, five and eight patients acquired at least one mutation in druggable genes during disease progression according to the OnkoKB (i.e., *SF3B1, ALK, ARID1A, KRAS, BRAF*) and TARGET (*ALK, ATR, DNMT3A, KRAS, BRAF, TP53*) databases (Table S2). Some of these mutations were acquired at the early stages of the disease (e.g., *KRAS*: first relapse), but there were cases with acquired mutations in more advanced disease (e.g., *BRAF*: third relapse, *KRAS*: third relapse, *SF3B1*: fourth relapse, *TP53*: sixth relapse, *ARID1A*: seventh relapse) (Table S2).

When focusing on mutations that were stable, expanded or acquired during anti-myeloma therapy, our data indicate that more than two-thirds of patients (26/30, 87% and 22/30, 73% according to the TARGET and OnkoKB databases, respectively) with relapsed or refractory MM carry at least one mutation in a druggable gene.

### 3.3. Copy Number Variants

**Figure 4:**
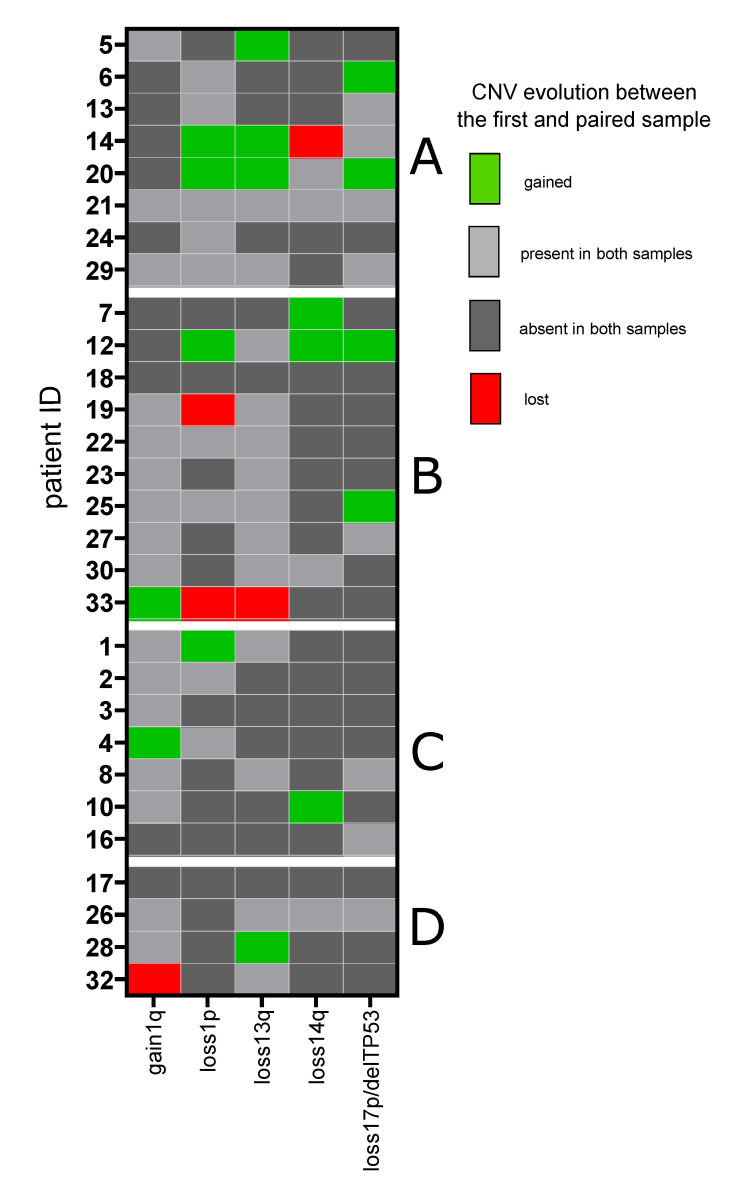
Evolution of copy number variants during multiple myeloma progression based on the next-generation sequencing data from the first and paired samples. Description: A—patients who presented a branching evolution of single-nucleotide variants with mutations lost and gained during multiple myeloma progression; B—patients who only acquired new single-nucleotide variants during multiple myeloma progression; C—patients who only lost single-nucleotide variants during multiple myeloma progression; D—patients with a stable profile of single-nucleotide variants during multiple myeloma progression.

Of the CNVs with prognostic significance, del1p (deletion of at least one of the following regions: 1p12, 1p22.1 and 1p32.3) was found in a total of 14 (47%) patients. In ten cases, del1p was detected in the first sample (including seven diagnostic samples), and in four cases, it was an acquired variant in the subsequent sample (Table 1). Twenty-one patients (70%) had gain 1q21—in 19 cases detected in the first sample (including nine diagnostic samples) and in the others (*n* = 2) as an acquired variant in paired sample. Two patients had gain 1q21 in the primary sample, which was lost in the following sample. However, in one of these cases, gain1q21 that had previously disappeared reappeared during the next MM progression. Del17p or delTP53 were found in a total of twelve (40%) patients, in eight cases in the first sample (including five diagnostic samples) and in four cases as an acquired variant in paired sample. Del13q and del14q were detected in the first sample in fourteen (47%) and six (20%) patients and were acquired during MM relapse in five (17%) and three (10%) additional cases, respectively. The evolution of selected CNVs during MM between the paired samples is summarized in Figure 4.

### 3.4. IgH Translocations

FISH data were available for the majority of samples, as 28 patients (93%) had FISH data from at least two samples. There were two triple-hit (del17p plus gain 1q21 plus del1p and t(4; 14) plus gain 1q21 plus del1p) and four double-hit events (gain 1q21 plus del1p, *n* = 2; gain1q21 plus del17p, *n* = 1; del1p plus del17p, *n* = 1) seen in diagnostic samples. In addition, two t(11; 14) were found in diagnostic samples—one as an isolated aberration and the other as a combined aberration with del17p. There were also four 14q32 rearrangements other than t(4; 14) and t(14; 16)—two as additional abnormalities to the double- and triple-hit events described above, one as an isolated aberration and one as a combined aberration with gain1q21.

In patients with only relapsed samples collected, t(4; 14) was found in four cases. It was combined with gain1q, del1p and del17p (*n* = 1), gain 1q21 and del17p (*n* = 1) and gain 1q21 (*n* = 2). Two patients had 14q32 rearrangement other than t(4; 14) and t(14; 16) detected, and all of them also had detectable chromosome 1 abnormalities (gain 1q21). In one case, an additional not specified 14q32 rearrangement was detected in the context of del17p plus gain 1q21 plus del1p.

### 3.5. Prognostic Significance of Genetic Abnormalities

We were unable to demonstrate the prognostic value of mutations in individual genes acquired during MM progression. Similarly, no significant correlations were found when patients were categorized according to the acquisition of mutations in genes involved in specific cellular processes such as (1) gene expression (*ARID1A*, *ATR*, *DNMT3A*, *KMT2B*, *KMT2C*, *KMT2D*, *KRAS*, *NCOR2*, *NOTCH3*, *PBRM1*, *RUNX1*, *SF3B1*, *SOX9*, *TP53* and *ZFHX*), (2) chromatin organization (*ARID1A*, *DNMT3A*, *KDM5A*, *KDM5C*, *KMT2B, KMT2C*, *KMT2D*, *NCOR2*, *NFKB2*, *PBRM1* and *PRDM9*), (3) NOTCH signaling (*NCOR2*, *NOTCH3*, *RUNX1* and *TP53*), (4) SUMOylation (*DNMT3A*, *NCOR2*, *NFKB2* and *TP53*) and (5) RAF activation (*BRAF* and *KRAS*).

**Figure 5:**
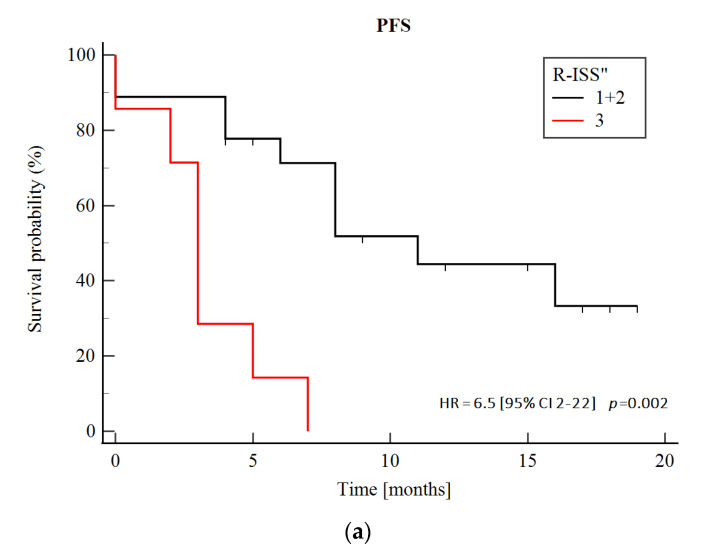

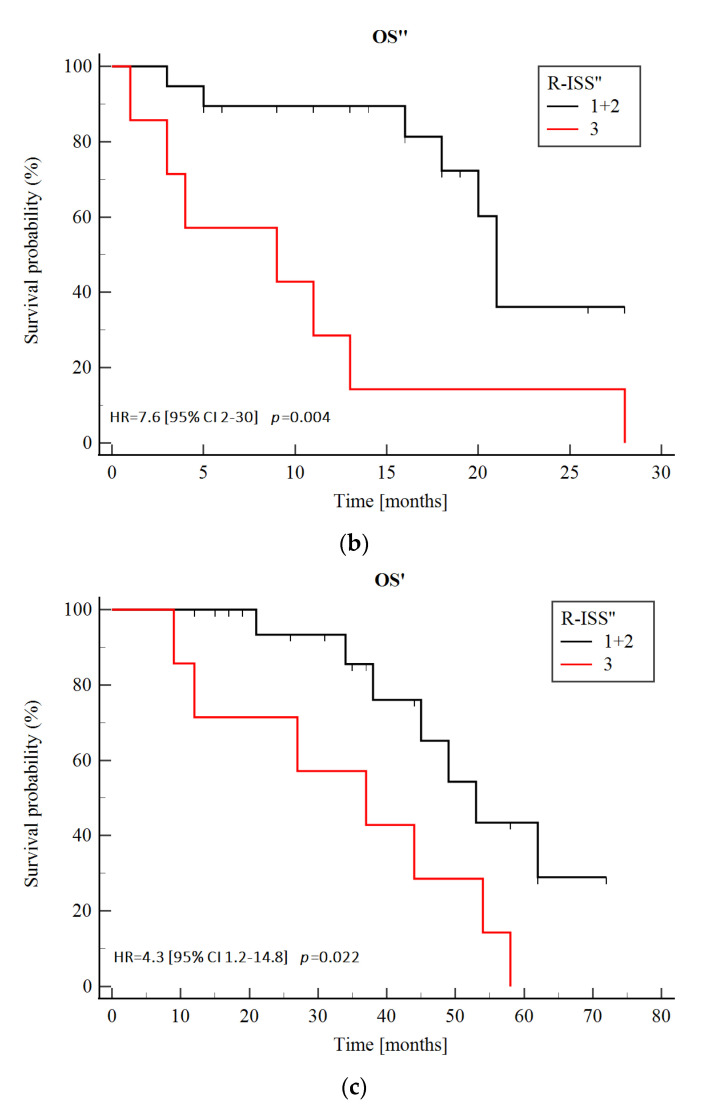
Kaplan–Meier survival curves for (**a**) PFS, (**b**) OS after the paired sample collection (OS″) and (**c**) OS after the first sample collection (OS′) according to the R-ISS″ (assessed at the time of the paired sample collection). The comparison of survival groups was assessed by log rank test (*p* values).

As there was a significant redistribution in the Revised International Staging System (R-ISS) risk categories between the first and paired samples (Table 1), in part due to CNV evolution, we tested the prognostic significance of R-ISS assessed at the time of paired sample collection (R-ISS″). In our cohort, a higher R-ISS″ (3 vs. 1 or 2) was significantly associated with an increased risk of subsequent progression (*p* = 0.026) and death (*p* = 0.016). Patients who had a higher R-ISS″ risk category (3 vs. 1 or 2) achieved a significantly shorter PFS (median, 3 and 11 months for patients with R-ISS″ 3 and 1 or 2, respectively; HR, 6.5; 95% CI, 2–22; *p* = 0.002). Furthermore, a higher R-ISS″ negatively affected both OS″ (median, 9 vs. 21 months for patients with R-ISS″ 3 and 1 or 2, respectively; HR, 7.6; 95% CI, 2–30; *p* = 0.004) and OS′ (median, 37 vs. 53 months for patients with R-ISS″ 3 and 1 or 2, respectively; HR, 4.3; 95% CI 1.2–14.8; *p* = 0.022) (Figure 5).

### 3.6. Biallelic Events

A total of eight biallelic events defined as a variant with VAF of at least 80% in at least one sample (excluding cases of amplified variants) across seven (23%) patients were detected (Table 2). Most patients were shown to have a biallelic event in two consecutive samples (patients 8, 25, 29 and 30), and two patients acquired two events between diagnosis and MM relapse (patients 7 and 14). Finally, a “second hit” during disease progression was observed in only one case (patient 28) (Table 2).

## 4. Discussion

Here, we used our custom gene panel for targeted DNA sequencing of tumor samples sequentially collected from 30 MM patients, including 19 and 11 patients with newly diagnosed MM and relapsed/refractory MM, respectively. Our cohort well reflected the heterogeneity of the MM patient population observed in real life, as we included patients with samples taken at diagnosis and first relapse but also those with samples taken after multiple lines of anti-myeloma therapy. In fact, thirteen patients (43%) in our cohort had at least one sample collected at the time of at least the third disease progression, which gives us relatively good insight into the clonal evolution of MM at very advanced stages. Although the cohort was small and heterogeneous, well-known clinical risk factors (i.e., refractoriness to proteasome inhibitors (PI), immunomodulatory drugs (IMiD) or alkylator agents) have been shown to retain significant prognostic significance in this cohort as well (Figure S2).

The molecular landscape of MM is complex, with prominent inter-patient genetic heterogeneity. Apart from a few well-defined recurrent structural variants (such as t(4; 14), t(14; 16), t(14; 20)) and CNV such as: del17p [1], MM is characterized by considerable SNV diversity with wide variations in the number of non-silent exonic mutations detected per patient across NGS studies, ranging from 15 to 46 (median, 31) [6], from 19 to 435 (median, 50) [2], from 21 to 488 (median, 52) [9] and from 59 to 226 (median, 152) [18] for newly diagnosed MM and from 22 to 333 (median, 77.5) for MM double-refractory to PI and IMiD [14]. Comparing with a previously published study by Corre et al. [8], in which a 246-gene panel was used for targeted sequencing of longitudinally collected samples from 43 homogeneously treated patients, we found a similar median number of variants per sample (median, 4 and 5 observed in our study vs. 4 and 5 reported by Corre et al., for diagnostic and relapsed sample, respectively) with a slightly larger range (range, 1–16 and 1–32 observed in our study vs. 0–12 and 0–12 reported by Corre at al., for diagnostic and relapsed sample, respectively). It should be emphasized here that we initially identified pathogenic variants using cancer-specific predictors rather than those designed for general purpose (e.g., PolyPhen2 [40] or SIFT [41]), as is the case in some NGS studies in MM [13,15,42].

Focusing on point mutations that survived selection by anti-myeloma therapy, we found that a significant proportion of SNV affected druggable genes. Although some of these variants decreased, most increased or were stable during MM progression. Moreover, there were examples of initially subclonal variants becoming clonal during disease progression. This finding is important in the context of personalized treatment, as the greatest clinical benefit can be achieved with targeted therapies acting on clonal mutations [43]. From a clinical point of view, it is also important that we identified variants of druggable genes persisting at very advanced stages of MM, including fifth, sixth and seventh relapse (Figure 3 and Figure S1) when patients usually suffer from a lack of valuable therapeutic options and targeted therapy may be of particular value.

Recently, studies using WES have shown that MM evolution during therapy follows one of several patterns (i.e., branching, linear or neutral evolution, differential clonal response and stable subclonal structure), with branching evolution being the most common [4,9,13,16,17]. In this context, four different patterns of progression were seen in our study, namely (1) branching evolution with at least one mutation appearing and another disappearing over time, (2) a mutation acquisition pathway characterized by the acquisition of at least one mutation over time, (3) a mutation loss pathway in which at least one variant was eradicated during treatment and (4) a stable mutational composition pathway in which no variant was acquired or lost during disease progression. In line with recent results by Jones et al. [16], we found that the evolutionary pattern of MM between paired samples is related to the depth of response to the treatment applied at that time.

Anti-myeloma therapy acts as a selection force for genetically distinct MM cells. Mutation gain observed during MM progression may therefore have occurred by selection and expansion of pre-existing very rare variants which could not be identified at the achieved sequencing depth in the initial sample (or were localized in non-sequenced tumor sites) or by the emergence of new variants due to the use of genotoxic drugs (e.g., melphalan, cyclophosphamide, bendamustine and doxorubicin), especially in the context of impaired DNA repair mechanisms [44].

Similarly to the shared mutations, five and eight patients acquired at least one mutation in druggable genes during disease progression according to the OnkoKB (i.e., *SF3B1*, *ALK*, *ARID1A*, *KRAS* and *BRAF*) and TARGET (i.e., *ALK*, *ATR*, *DNMT3A*, *KRAS*, *BRAF* and *TP53*) databases. These were both clonal and subclonal mutations occurring at early stages of the disease (i.e., first relapse), as in heavily pre-treated patients (i.e., third, fourth, sixth and seventh relapse). An important observation is that targetable genetic alteration can occur at very advanced stages of MM. Patients then often suffer from a lack of treatment options, so therapy targeting these alterations can be of great value. Therefore, evaluation of mutations in druggable genes should also be considered at the late stages of the disease.

Like others [5,8,15], we found MAPK/ERK pathway most commonly affected in MM. Mutations in at least one of the MAPK/ERK genes were present in 16 out of 30 patients. Surprisingly, we did not find *NRAS* mutations in our final (post-filtered) data set. This may be because we had a larger representation of patients with *KRAS* mutations (47% of patients) compared to previous studies [8,15], and *NRAS* and *KRAS* mutations have been shown to be mutually exclusive [5]. Although in some cases, mutations in MAPK/ERK pathway genes decreased over time, their eradication was observed in only two cases, highlighting the important contribution of MAPK/ERK activity not only in the development of newly diagnosed MM but also in driving MM progression. The decrease in the prevalence of *KRAS* mutations observed in some samples derived from relapsed/refractory MM patients in our study indicates that these mutations did not provide the cells carrying them with a selection advantage over other myeloma cells during relapse. It is likely that *KRAS*-mutated cells survived the selection pressure imposed by combined anti-myeloma therapy (at the level of residual disease) but, with the evolution of MM, were dominated by other more aggressive clones at the time of overt relapse. This once again highlights the complexity of the processes driving resistance to anti-myeloma therapy. An alternative explanation is that the genetic structure of MM at two different sites may be different and clonal abnormalities at one site may be subclonal at another [2].

Deregulation of the TP53 pathway is important in the development of MM, and del17p/TP53 inevitably defines high-risk disease in both primary and relapsed/refractory settings [45]. We had a high representation of patients with affected TP53 pathway, as *TP53* deletions and mutations were found in a total of 12 (40%) and 5 patients (17%), respectively. Most of the detected *TP53* mutations were subclonal (67%), and all of them, as previously noted [46], appeared in patients who also carried *TP53* deletion (Table S3). The high prevalence of TP53 pathway dysregulation (45% of patients) was also shown in a study of 42 patients resistant to both PI and IMiD [14]. As recently demonstrated, biallelic *TP53* inactivation provides an extremely poor prognosis in MM patients [47]. In our cohort, we had one patient who acquired such a “double-hit” event during MM progression. Surprisingly, this patient achieved a complete remission from the second-line treatment with daratumumab–bortezomib–dexamethasone combo, which has been sustained to date (15 months).

As many as 50% of our patients (15/30) carried persistent or acquired mutations in at least one epigenetic regulator, which is in line with the increasing role attributed to epigenetics deregulation in MM development and progression [48]. In our cohort, mutations in epigenes previously described in MM (*ARID1A*, *DNMT3A*, *KDM5C*, *KMT2B* and *KMT2C*) [5,7,8,15,22,23,24] as well as in novel epigenetic regulators (e.g., *KDM5A*, *KMT2A*, *KMT2D*, *PRDM9* and *PBRM1*) appeared when the disease progressed.

We had 23% and 27% of patients IMiD-refractory before the first and between the first and paired samples, respectively (Table 1). Using a filtering strategy as described in the methodology, among the genes potentially associated with IMiD-resistance (*CRBN*, *DDB1*, *RBX1*, *CUL4B*, *IKZF1* and *IKZF3*) [49,50], we found only *RBX1* affected in one patient (refractory to lenalidomide and pomalidomide). Similarly, for genes potentially associated with PI-resistance (*PSMB5*, *PSMB8*, *PSMB9*, *PSMD1*, *PSMG2* and *XBP1*) [50], we found an acquired Q240H *XBP1* mutation (in patient refractory to carfilzomib and ixazomib). Collectively, our data show that the acquisition of point mutations in the above-mentioned genes is not a leading mechanism of resistance to IMiD and PI, confirming previous observations [14].

It is known that the mutational complexity of MM differs significantly between patients, and there were hypermutated cases with extensive subclonal architecture observed across studies [13,14]. In our cohort, we had two cases that acquired more than 10 mutations between consecutive samples. One patient (patient 30, Figure S1) had slowly progressive MM with advanced bone disease. In contrast, the other one (patient 21, Figure 3) experienced dynamic progression of MM with the involvement of multiple extramedullary sites, including the pericardium.

We and others have previously shown that the evolution of cytogenetic aberrations over time has prognostic significance in MM [51,52,53]. Recently, Yan et al. performed a single-cell analysis using quantitative multi-gene FISH on 129 longitudinally sampled specimens from 57 MM patients confirming that patients with stable cytogenetic structure achieve significantly longer survival than patients with evolutionary dynamics [54]. In our dataset, we identified only two patients (7%) with a stable CNV profile (Table S1). Moreover, all but one of the four patients with a stable SNV profile acquired or simultaneously gained and lost CNV during disease progression (Table S3). Collectively, this again shows that genetic events involving multiple genes, such as CNV, play a far greater role than single-point mutations in MM pathogenesis and progression [4,5,14,22].

Although we had a heterogeneous group of patients treated non-uniformly, we demonstrated a high clinical utility of R-ISS reassessment at MM progression. In our cohort, patients with a higher R-ISS risk category based on parameters obtained at the time of the paired sample collection achieved a significantly shorter median PFS as well as OS″ and OS′ (Figure 5). This once again suggests the importance of dynamic risk assessment during the course of MM and cytogenetics reassessment at the time of disease progression.

Our results, although informative, should be viewed in the context of the study limitations arising in particular from the methodology. Targeted sequencing can provide high-depth sequencing data in a short period of time, enabling rapid identification of prognostic or predictive genetic factors and actionable gene mutations representing targets for personalized therapies. However, tracking changes in an arbitrarily defined set of genes limits insight into the actual genetic structure of a tumor and its evolution over time. Using a tumor-only sequencing approach further limits the ability to reliably distinguish the origin (germinal vs. somatic) of identified genetic variants [55]. The lack of matched germline DNA in our study implies that the somatic origin of all identified variants, especially those with VAF ≥ 40% in each sample, might be uncertain [56]. Moreover, it should be noted that MM is characterized by marked spatial genetic heterogeneity [2]; therefore, sequencing a sample from a single tumor site underestimates its mutational complexity [43].

## 5. Conclusions

In summary, our custom gene panel allowed us to successfully track the clonal evolution of MM in a heterogenous cohort of patients that well mimics the real-life population. In our cohort, we had a good insight into genetic changes occurring at very advanced stages of MM, and we were able to identify point mutations in druggable genes in a clinically relevant proportion of cases. We show that there is a link between the depth of response to anti-myeloma therapy and the evolutionary pattern of the disease. Additionally, we confirmed that cytogenetic reassessment to redefine R-ISS at the time of disease progression is of high clinical value.

## Figures and Tables

**Table 1:** Patient and disease characteristics.

Parameter	At the Time of the 1st Sample Collection (*n* = 30)		At the Time of the Paired Sample Collection (*n* = 30)
**Age (years), median (range)**	65 (50–77)		67 (50–82)
**Female and male sex**	13 (43%), 17 (57%)		
**ECOG score**			
0–1	23 (77%)		23 (77%)
≥2	5 (16%)		5 (16%)
Not reported	2 (7%)		2 (7%)
**ISS staging**			
I	7 (23%)		8 (27%)
II	13 (43%)		4 (13%)
III	9 (30%)		14 (47%)
Not reported	1 (3%)		4 (13%)
**R-ISS staging**			
I	3 (10%)		6 (20%)
II	23 (77%)		13 (44%)
III	3 (10%)		7 (23%)
Not reported	1 (3%)		4 (13%)
**Cytogenetics/Copy number variants**			
t(4; 14)	5 (17%)		
14q32 rearrangement, other ^a^	8 (27%)		
14q32 rearrangement, not specified	1 (3%)		
	Detected in the 1st sample (*n* = 30)		Acquired in the paired sample (*n* = 30)
del1p	10 (33%)		4 (14%)
gain1q21	19 (63%)		4 (14%)
del17p/17 monosomy	8 (27%)		4 (14%)
del14q	6 (20%)		3 (10%)
del13q	13 (43%)		5 (17%)
**Lines of therapy—in total,** **median (range)**	4 (1–8)		
**Lines of therapy, median (range)**	Before the 1st sample	Between the 1st and paired samples	After the paired sample
	3 (1–6) ^b^	1 (1–3)	1 (0–3)
**Multiple myeloma therapy**			
Exposure to PI (i.e., bortezomib, carfilzomib, ixazomib)	8 (27%)	24 (80%)	12 (40%)
PI-refractoriness	1 (3%)	11 (37%)	7 (23%)
Exposure to IMiD (i.e., thalidomide, lenalidomide, pomalidomide)	10 (33%)	15 (50%)	10 (33%)
IMiD-refractoriness	7 (23%)	8 (27%)	4 (13%)
Double-refractoriness to IMiD and PI	0	4 (13%)	1 (3%)
Exposure to cytotoxic agents (e.g., bendamustine, cyclophosphamide, doxorubicin, vincristine, melphalan)	8 (27%)	20 (67%)	10 (33%)
Refractory to cytotoxic agents	2 (7%)	7 (23%)	6 (20%)
Triple-refractoriness to IMiD, PI and alkylators	0	2 (7%)	0
Autologous stem cell transplantation	8 (27%)	10 (33%)	1 (3%)
Allogeneic stem cell transplantation	1 (3%)	1 (3%)	1 (3%)

^a^, 14q32 rearrangement other than t(4; 14) and t(14; 16). ^b^, only for patients without available diagnostic samples. Abbreviations: ECOG—Eastern Cooperative Oncology Group; IMiD—immunomodulatory drugs; ISS—International Staging System; PI—proteasome inhibitors; R-ISS—Revised International Staging System.

**Table 2:** Biallelic events detected in the first and paired samples.

Patient ID	Sample Type	Gene	cDNA	ACMG Classification According to Varsome [36]
8	Dx and PD	*EP300*	c.736A>G	Uncertain significance
8	Dx and PD	*CDKN1B*	c.180G>A	Pathogenic
14	PD	*TP53*	c.711G>A	Pathogenic
25	PD and PD	*ATM*	c.6833T>A	Uncertain significance/minor pathogenic evidence
28	PD	*BRCA2*	c.8182G>A	Uncertain significance
29	Dx and PD	*USP9X*	c.2939C>G	Uncertain significance/minor pathogenic evidence
30	Dx and PD	*FGFR3*	c.2204G>A	Likely pathogenic
7	PD	*FAT4*	c.10571G>A	Uncertain significance

Abbreviations: ACMG—American College of Medical Genetics; cDNA—complementary DNA; CNV—copy number variant; Dx—diagnosis; PD—progressive disease; VAF—variant allele frequency.

## Data Availability

The data presented in this study are available on request from the corresponding author.

## Supplementary Materials

The following supporting information can be downloaded at: https://www.mdpi.com/article/10.3390/biomedicines10071674/s1, Figure S1: Evolution of single-nucleotide variants during multiple myeloma progression in patients with more than two samples collected; Figure S2: Prognostic significance of clinical risk factors in the study cohort; Table S1: Gene panel; Table S2: Summary of identified gene variants; Table S3: Summary of the genetic profile of each subsequent sample.
