# Supplementary material for: Tracking Clonal Evolution of Multiple Myeloma Using Targeted Next-Generation DNA Sequencing
Source: Biomedicines. 2022 Jul 12;10(7):1674. doi: 10.3390/biomedicines10071674 (PMC9313382; doi:10.3390/biomedicines10071674)
Supplement: Supplementary file 1 [file biomedicines-10-01674-s001.zip › Figure S1.pdf]

Figure S1. Evolution of single nucleotide variants during multiple myeloma progression in patients with more than two samples collected.

Abbreviations: B, biochemical; C, clinical; ND, new diagnosis; P, patient; R, relapse.

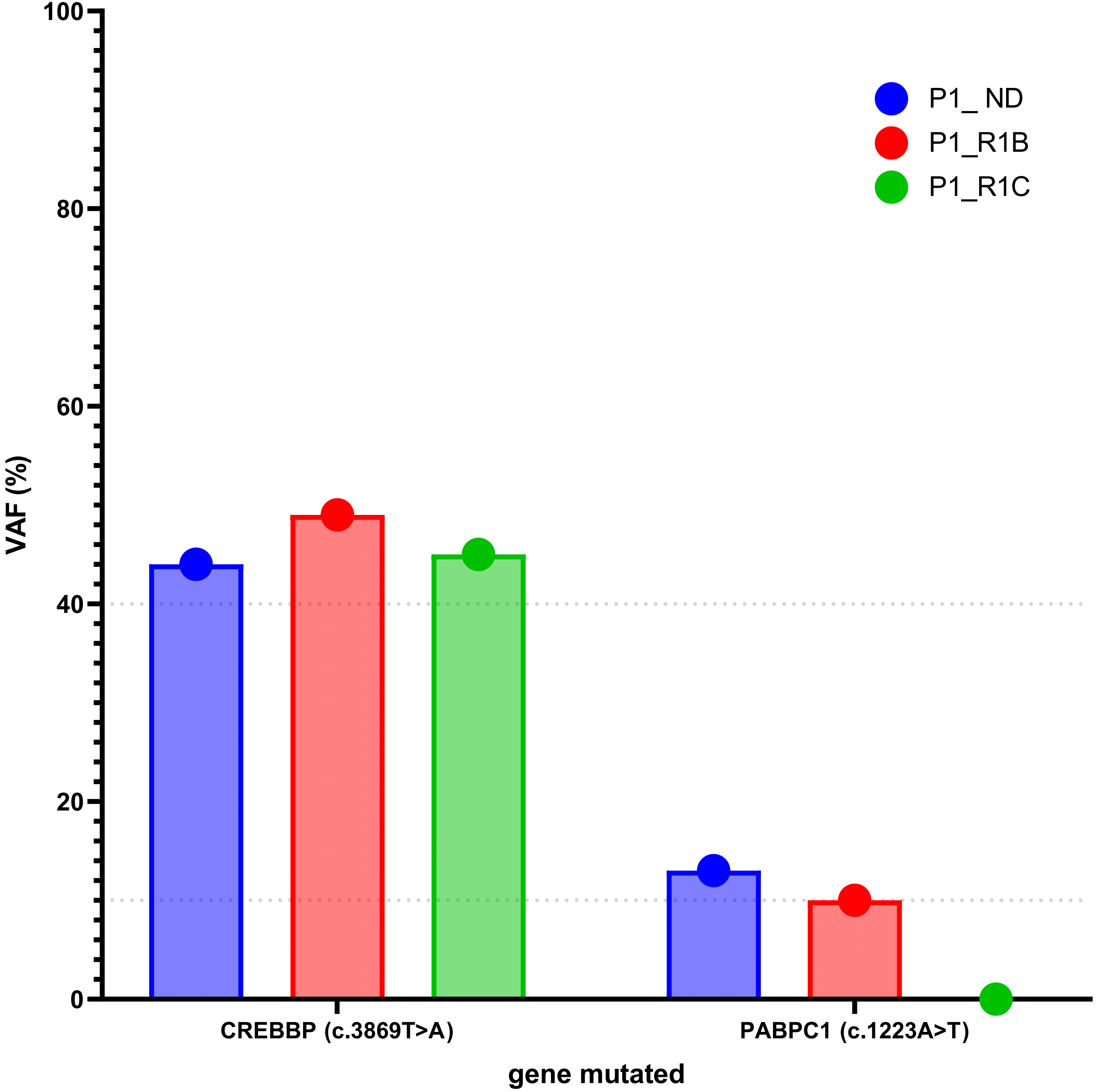

100
80
60
40
20
0
VAF (%)
P1_ ND
P1_R1B
P1_R1C
CREBBP (c.3869T>A)
PABPC1 (c.1223A>T)
gene mutated

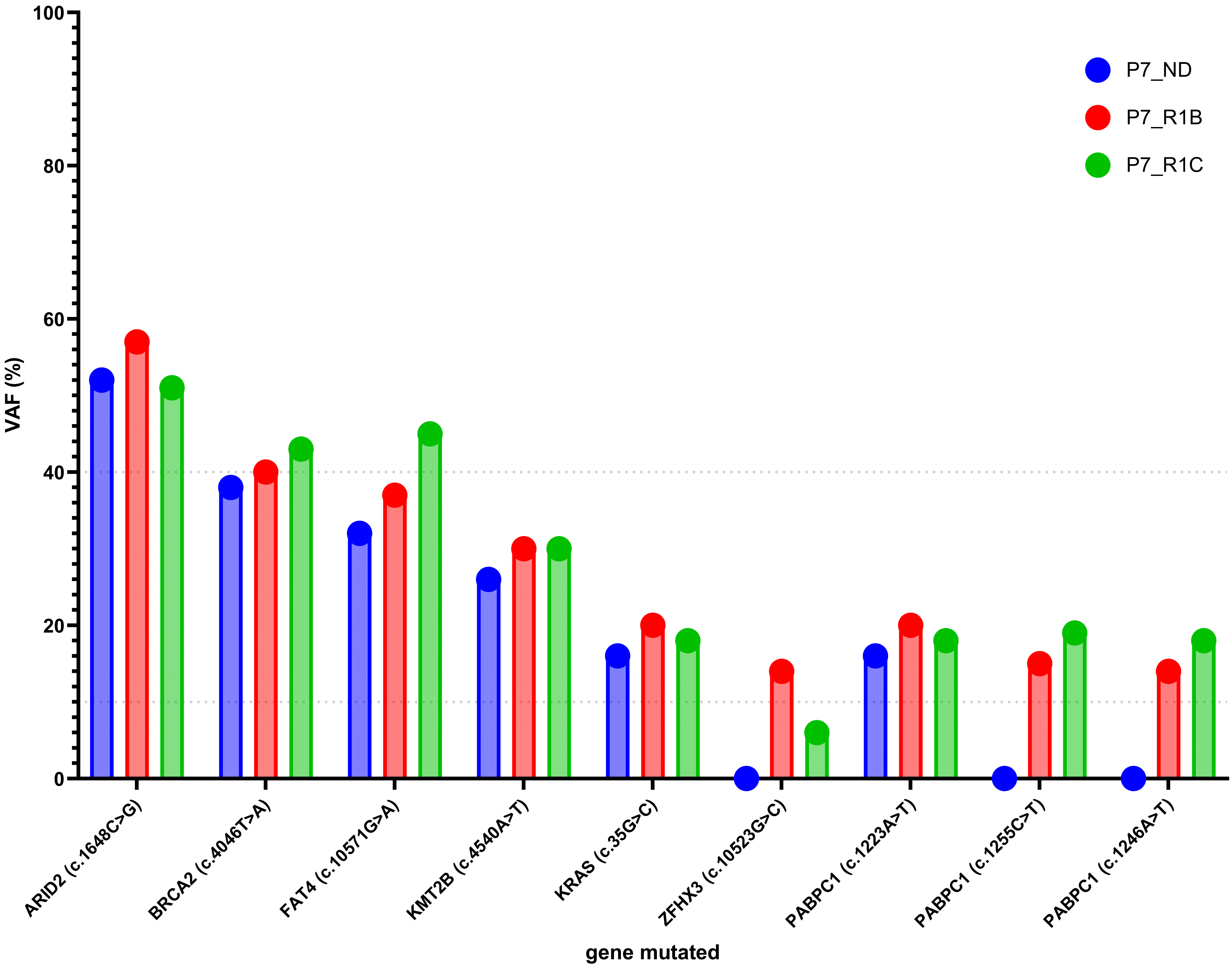

100
80
60
40
20
0
VAF (%)
P7_ND
P7_R1B
P7_R1C
ARID2 (c.1648C>G)
BRCA2 (c.4046T>A)
FAT4 (c.10571G>A)
KMT2B (c.4540A>T)
KRAS (c.35G>C)
ZFHX3 (c.10523G>C)
PABPC1 (c.1223A>T)
PABPC1 (c.1255C>T)
PABPC1 (c.1246A>T)
gene mutated

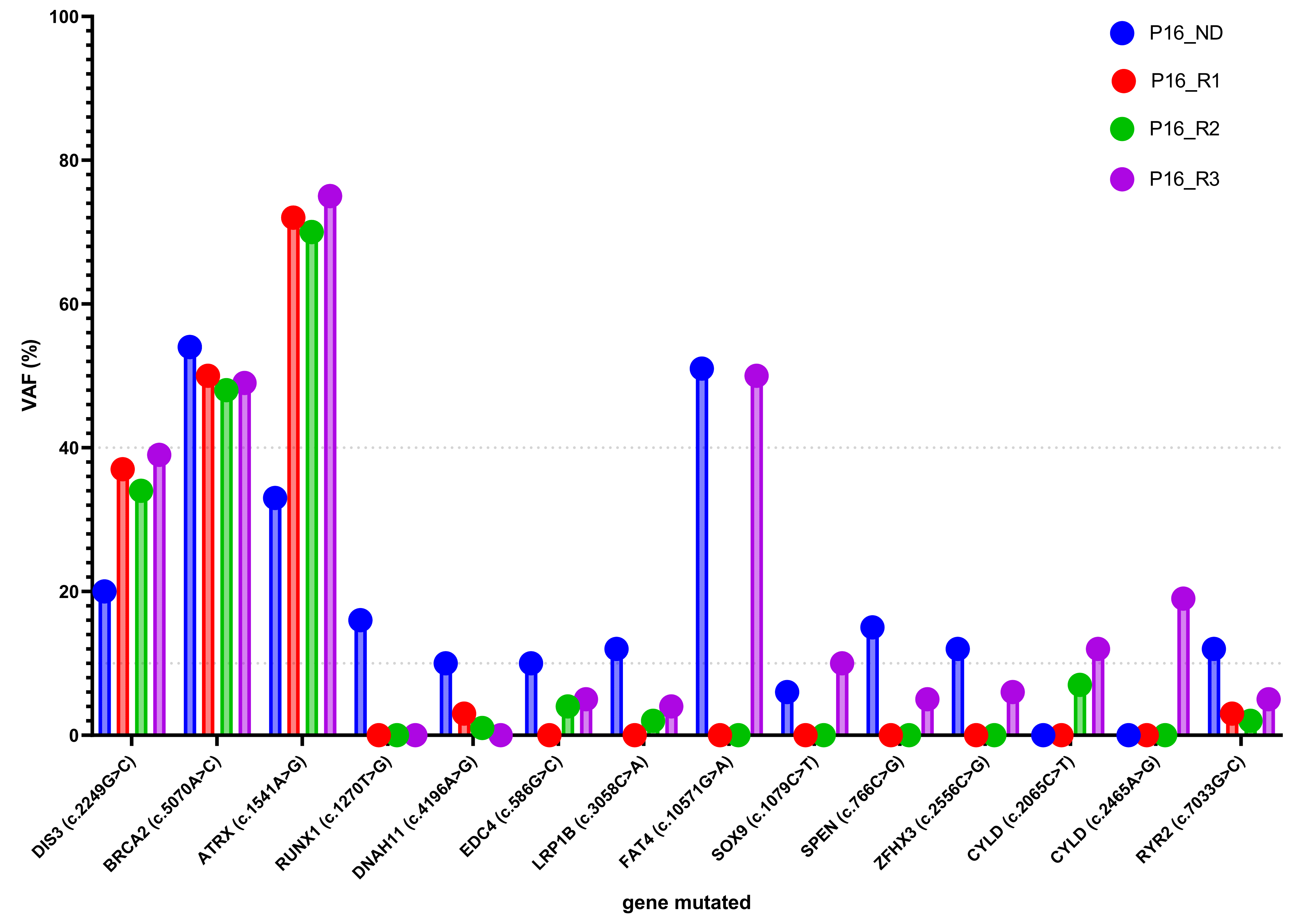

100
80
60
40
20
0
VAF (%)
P16_ND
P16_R1
P16_R2
P16_R3
DIS3 (c.2249G>C)
BRCA2 (c.5070A>C)
ATRX (c.1541A>G)
RUNX1 (c.1270T>G)
DNAH11 (c.4196A>G)
EDC4 (c.586G>C)
LRP1B (c.3058C>A)
FAT4 (c.10571G>A)
SOX9 (c.1079C>T)
SPEN (c.766C>G)
ZFHX3 (c.2556C>G)
CYLD (c.2065C>T)
CYLD (c.2465A>G)
RYR2 (c.7033G>C)
gene mutated

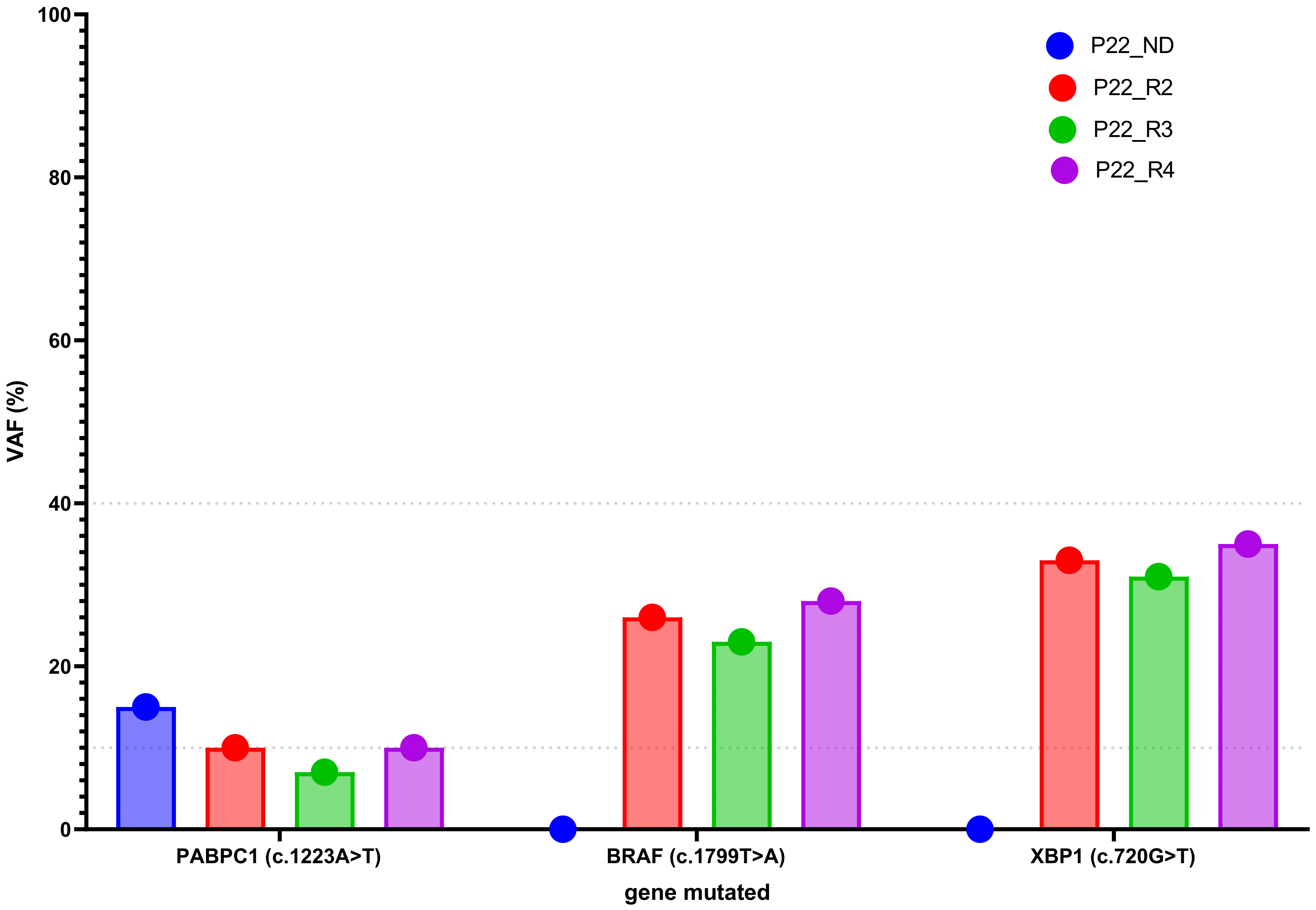

100
80
60
40
20
0
VAF (%)
P22_ND
P22_R2
P22_R3
P22_R4
PABPC1 (c.1223A>T)
BRAF (c.1799T>A)
XBP1 (c.720G>T)
gene mutated

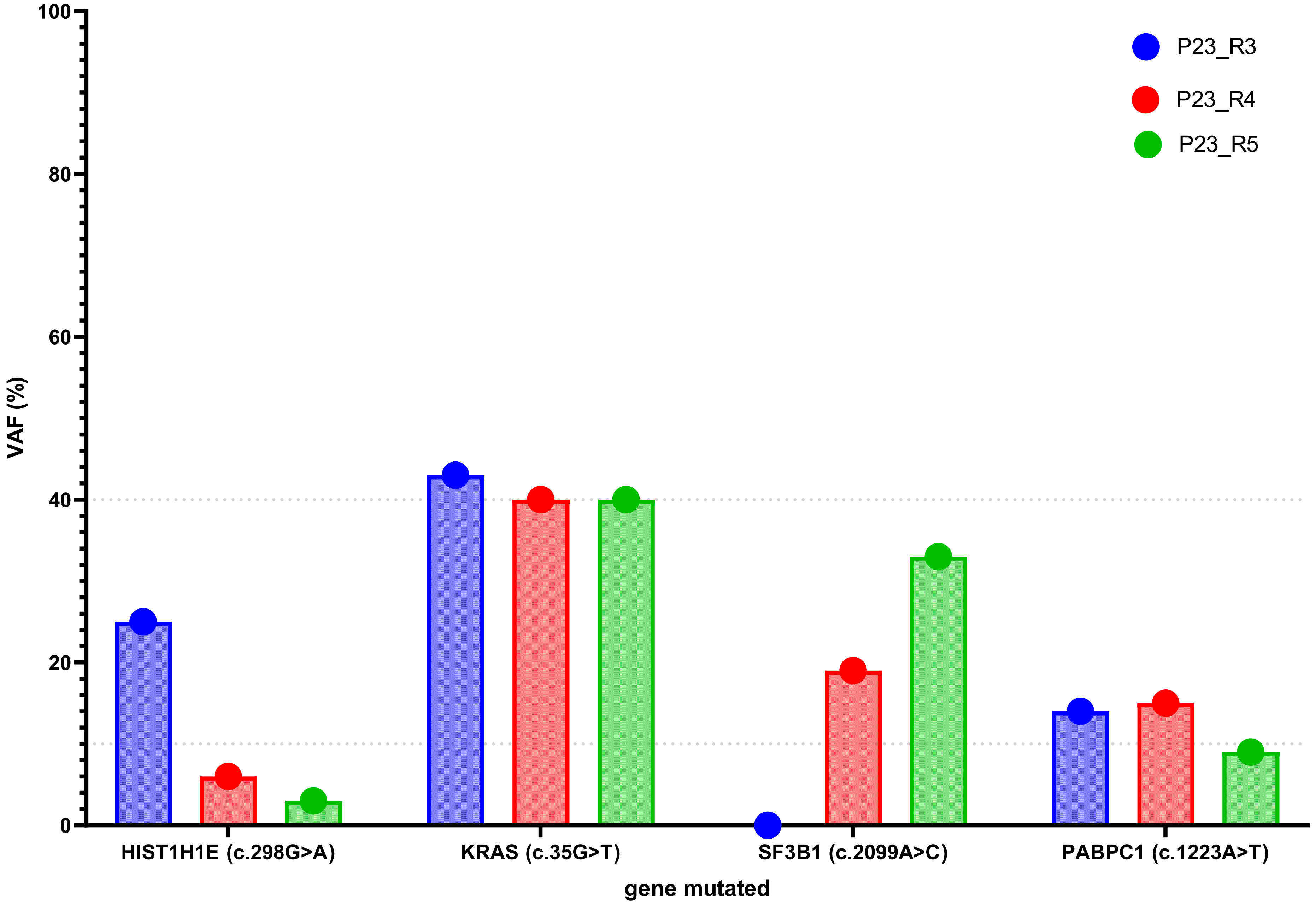
100
80
60
40
20
0
VAF (%)
P23_R3
P23_R4
P23_R5
HIST1H1E (c.298G>A)
KRAS (c.35G>T)
SF3B1 (c.2099A>C)
PABPC1 (c.1223A>T)
gene mutated

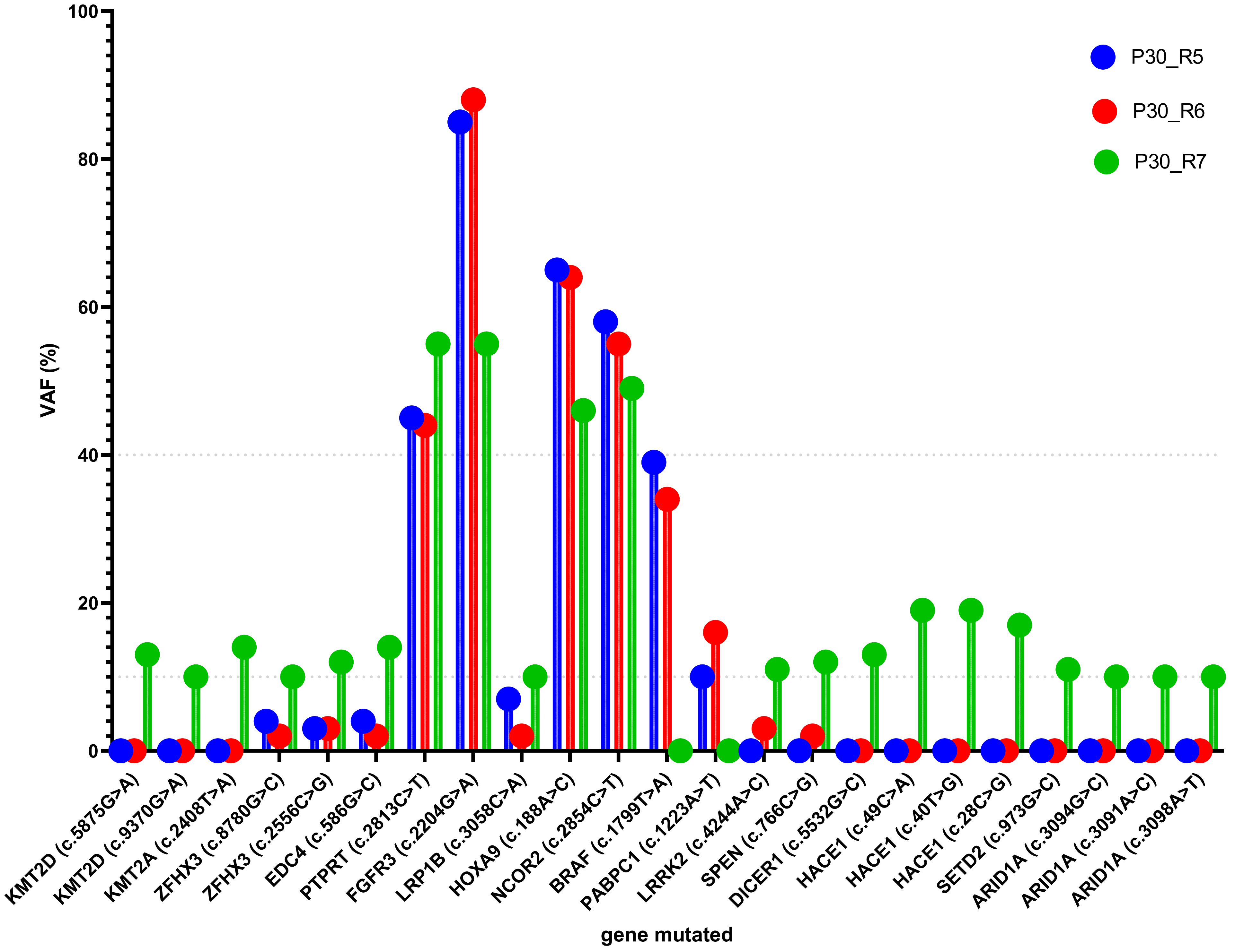

VAF (%)
100
80
60
40
20
0
P30_R5
P30_R6
P30_R7
KMT2D (c.5875G>A)
KMT2D (c.9370G>A)
KMT2A (c.2408T>A)
ZFHX3 (c.8780G>C)
ZFHX3 (c.2556C>G)
EDC4 (c.586G>C)
PTPRT (c.2813C>T)
FGFR3 (c.2204G>A)
LRP1B (c.3058C>A)
HOXA9 (c.188A>C)
NCOR2 (c.2854C>T)
BRAF (c.1799T>A)
PABPC1 (c.1223A>T)
LRRK2 (c.4244A>C)
SPEN (c.766C>G)
DICER1 (c.5532G>C)
HACE1 (c.49C>A)
HACE1 (c.40T>G)
HACE1 (c.28C>G)
SETD2 (c.973G>C)
ARID1A (c.3094G>C)
ARID1A (c.3091A>C)
ARID1A (c.3098A>T)
gene mutated

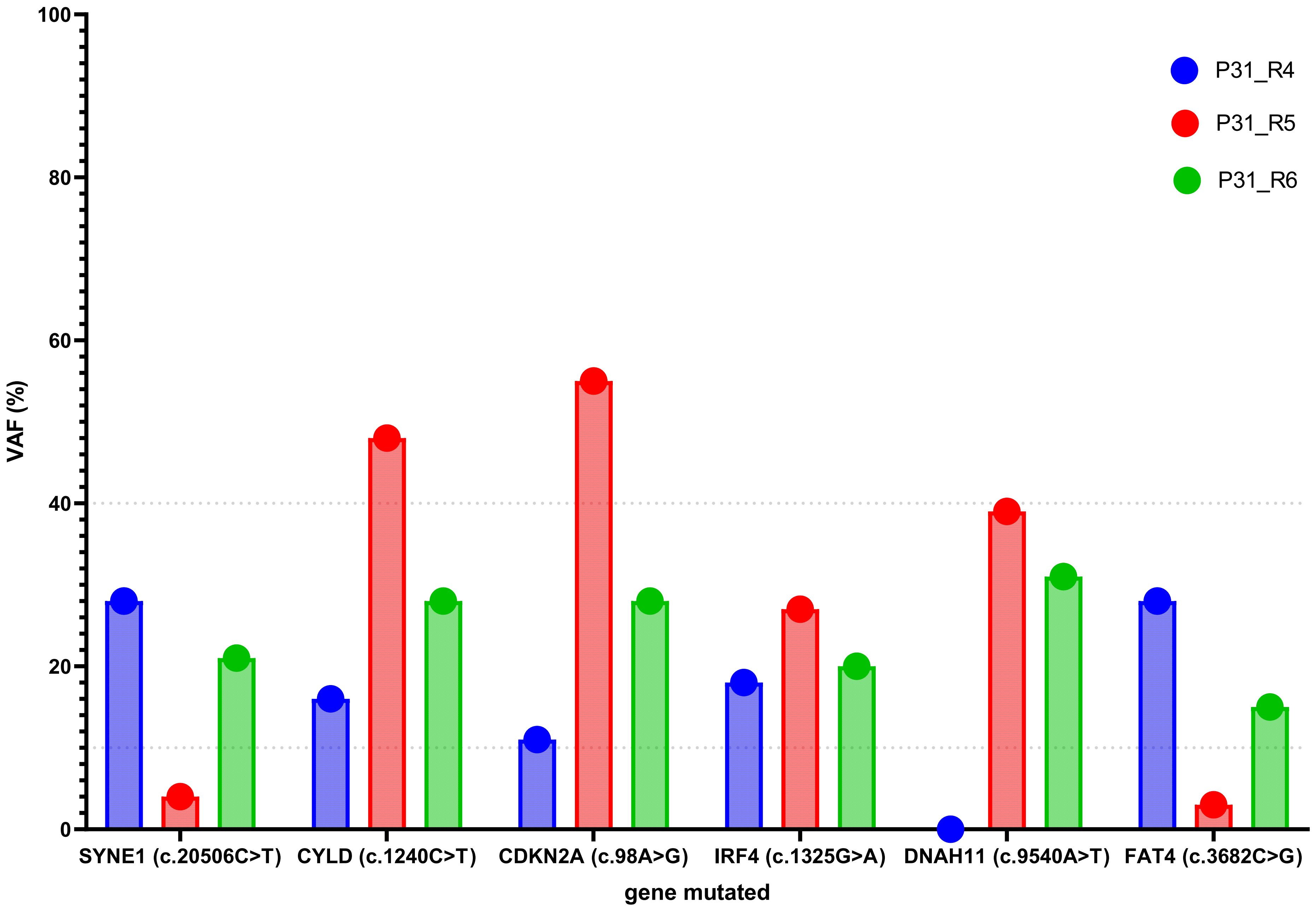

100
80
60
40
20
0
VAF (%)
P31_R4
P31_R5
P31_R6
SYNE1 (c.20506C>T)
CYLD (c.1240C>T)
CDKN2A (c.98A>G)
IRF4 (c.1325G>A)
DNAH11 (c.9540A>T)
FAT4 (c.3682C>G)
gene mutated
