# Supplementary material for: Tracking Clonal Evolution of Multiple Myeloma Using Targeted Next-Generation DNA Sequencing
Source: Biomedicines. 2022 Jul 12;10(7):1674. doi: 10.3390/biomedicines10071674 (PMC9313382; doi:10.3390/biomedicines10071674)
Supplement: Supplementary file 1 [file biomedicines-10-01674-s001.zip › Figure S2.pdf]

Figure S2. Prognostic significance of clinical risk factors in the study cohort. The comparison of survival groups was assessed by log rank test (*p* values).

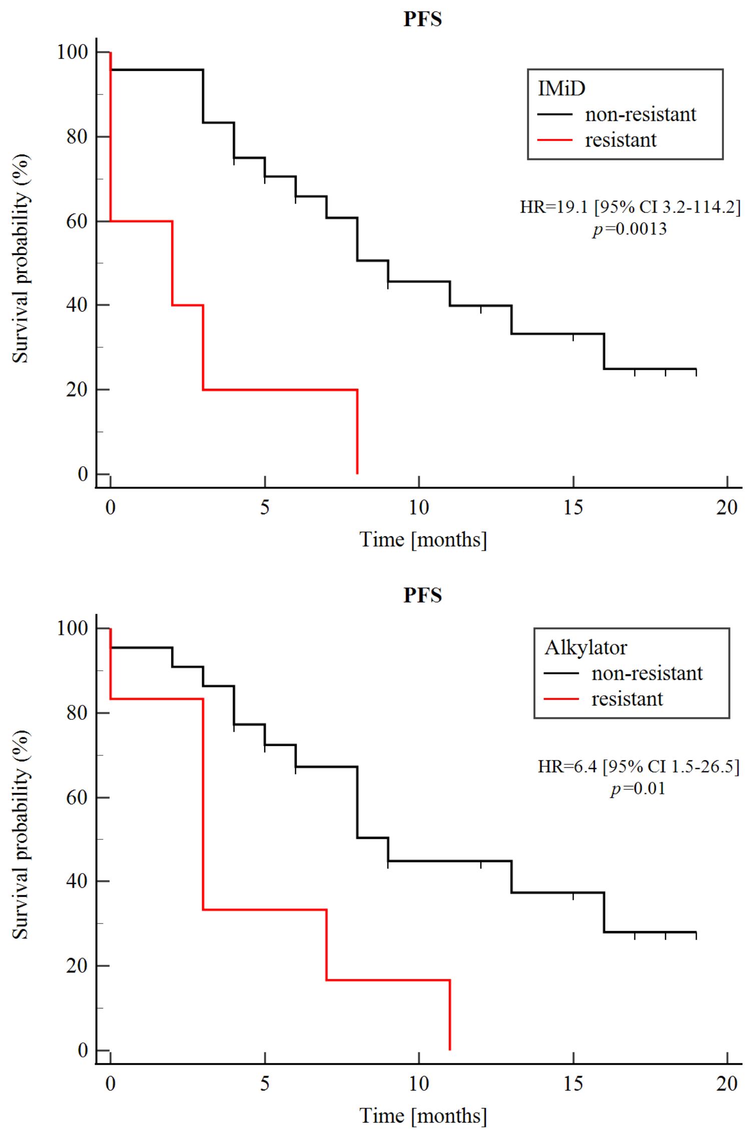

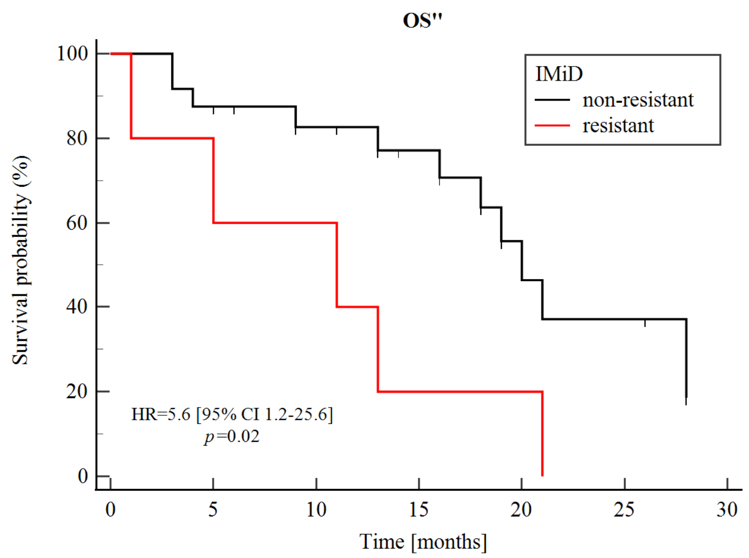
OS''
IMiD
non-resistant
resistant
HR=5.6 [95% CI 1.2-25.6]
p=0.02
Survival probability (%)
Time [months]
100
80
60
40
20
0
0
5
10
15
20
25
30

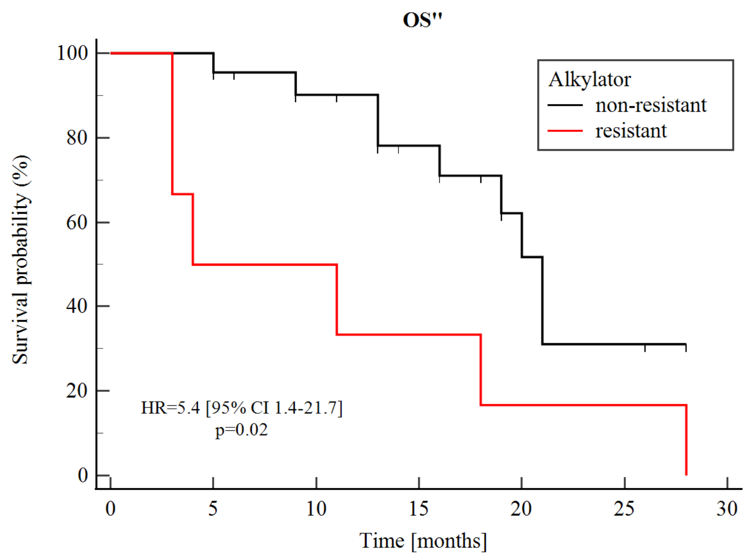
OS''
Alkylator
non-resistant
resistant
HR=5.4 [95% CI 1.4-21.7]
p=0.02
Survival probability (%)
Time [months]
100
80
60
40
20
0
0
5
10
15
20
25
30

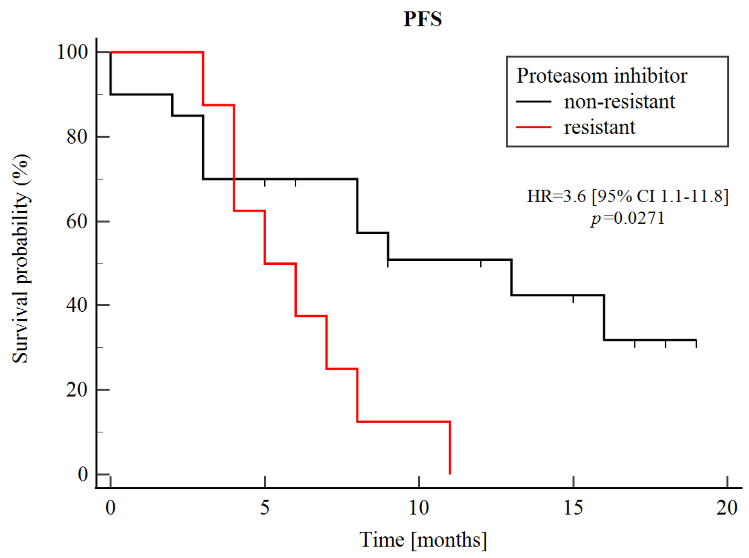
PFS
Proteasom inhibitor
non-resistant
resistant
HR=3.6 [95% CI 1.1-11.8]
p=0.0271
Survival probability (%)
Time [months]
100
80
60
40
20
0
0
5
10
15
20
