# Supplementary material for: Tracking Clonal Evolution of Multiple Myeloma Using Targeted Next-Generation DNA Sequencing
Source: Biomedicines. 2022 Jul 12;10(7):1674. doi: 10.3390/biomedicines10071674 (PMC9313382; doi:10.3390/biomedicines10071674)
Supplement: Supplementary file 1 [file biomedicines-10-01674-s001.zip › Table S3.pdf]

Table S1. Summary of the genetic profile of each subsequent sample.

a) Cases with diagnostic sample (a total of 2 or 3 samples)

Patient ID	Data type	1st sample	2nd sample	3rd sample
1	-	diagnosis	PD1b	PD1
	SNV	*CREBBP*, *PABPC1*	*CREBBP*, *PABPC1*	*CREBBP*
	CNV	+5, gain 1q, gain 6p, gain 15q, loss 13q, loss 22q	+19, gain 1q, gain 6p, gain 15q, loss 1p22.1, loss 2q (partial), loss 6q (partial), loss 13q, loss 20q, loss 22q	+5, +19, gain 1q, gain 6p, gain 15q, loss 1p22.1, loss 2q (partial), loss 6q (partial), loss 13q, loss 22q
	FISH	No detectable 14q32 translocations	No detectable 14q32 translocations	No detectable 14q32 translocations
2	-	diagnosis	PD1	-
	SNV	*RET*, *KRAS* , *CDKN2C*	*RET*, *KRAS*	-
	CNV	gain 1q21, +3, +5, +7, +9 (partial), gain 15q, gain 19p, +21 (partial), loss 1p, loss 16q (partial), loss 18q, loss 22q	gain 1q, gain 1p (partial), +3, +5, gain 6p22.1, +7, +9 (partial), gain 11q24, gain 15q, gain 19p, +21 (partial), loss 1p12, loss 18q, loss 22q	-
	FISH	No detectable 14q32 translocations	No detectable 14q32 translocations	-
3	-	diagnosis	PD1	-
	SNV	BRAF , FANCA, LRP1B	BRAF , FANCA	-
	CNV	gain 1q, +3, gain 5q (partial), +6 (partial), gain 15q, +19, +21 (partial), loss 6q (partial), -8, -20, loss 22q	gain 1q, gain 1p (partial), +3, +6 (partial), gain 15q, +19, +21 (partial), -8, -20	-
	FISH	No detectable 14q32 translocations	No detectable 14q32 translocations	-
4	-	diagnosis	PD1	-
	SNV	KRAS, MLH1, APC, PABPC1	KRAS, MLH1 , APC	-
	CNV	+5, +11, gain 15q, loss 1p (partial), loss 6q, loss 18q	gain 1q, +5, gain 6p22.1, +11, gain 15q, gain 20q (partial), loss 1p (partial), -6 (partial), loss 18q	-
	FISH	No detectable 14q32 translocations	No detectable 14q32 translocations	-
5	-	diagnosis	PD1	-
	SNV	DNAH5, KRAS, JAK3 , SAMHD1 , PABPC1	DNAH5, KRAS, JAK3, SAMHD1 , KMT2C	-
	CNV	gain 1q, gain 6p22.1, gain 8q (partial), loss 16q (partial)	+1, gain 6p22.1, gain 8q (partial), gain 20q (partial), loss 13q (partial), loss 20p (partial),	-
	FISH	Translocation 14q32 other than t(4;14) and t(14;16)	Translocation 14q32 other than t(4;14) and t(14;16)	-
6	-	diagnosis	PD1	-
	SNV	RYR2, ARID4B , NCOR2	RYR2, ARID4B, PABPC1 , KRAS , LRP1B	-
	CNV	+3, +5, gain 6p22.1, +9, +11, gain 15q, +19, loss 1p (partial), loss 16q (partial), -18	+3, +5, gain 6p22.1, +9, +11, gain 15q, +19, loss 1p (partial)	-
	FISH	No detectable 14q32 translocations	No detectable 14q32 translocations, TP53 deletion	-
7	-	diagnosis	PD1b	PD1
	SNV	PABPC1 (c.1223A>T) , KRAS , ARID2, IDH2, BRCA2 , KMT2B	PABPC1 (c.1223A>T) , KRAS , ARID2, BRCA2, KMT2B , PABPC1 (c.1246A>T) , PABPC1 (c.1255C>T) , ZFHX3	PABPC1 (c.1223A>T) , KRAS, ARID2, BRCA2, KMT2B , PABPC1 (c.1246A>T) , PABPC1 (c.1255C>T) , ZFHX3 , FAT4
	CNV	+3, +6, +7, +9, +11, gain 15q, +18, +19, +21 (partial)	+3, +6, +7, +9, +11, gain 15q, +18, +19, +21 (partial)	+3, +6, +7, +9, +11, gain 15q, +18, +19, +21 (partial), loss 14q, loss 16q
	FISH	No detectable 14q32 translocations	No detectable 14q32 translocations	No detectable 14q32 translocations
8		diagnosis	PD1	-
	SNV	NOTCH2, EP300, CDKN1B, TBX3, PABPC1	NOTCH2, EP300, CDKN1B, TBX3	-
	CNV	gain 1q (partial), +7, gain 8q, gain 9q (partial), gain 19p, loss 6q, loss 8p, -12, loss 13q, -16, loss 17p (partial), loss 19q (partial), loss 22q	+1, +7, gain 6p22.1, gain 8q, gain 9p, gain 19p, gain 21q, loss 2q, loss 3q26, -4, -6 (partial), loss 8p, -10, -12, loss 13q, loss 16q, loss 17p (partial), loss 19q (partial), loss 22q	-
	FISH	Translocation 14q32 other than t(4;14) and t(14;16), TP53 deletion	No data available	-
10	-	diagnosis	PD1	-

	SNV	FAT4, KRAS , ARID1A , RYR2 , ATR , KMT2D , EDC4 , MAP3K14, LRP1B (c.3058C>A) , MAN2C1 , ZFHX3 (c.10523G>C), HUWE1 , LRP1B (c.3075T>G) , NCOR2 , ZFHX3 (c.10520G>C) , EP300	FAT4, ATM, KRAS, ARID1A , RYR2 , ATR , KMT2D , EDC4 , MAP3K14	-
	CNV	gain 1q, gain 6p22.1, gain 15q	gain 1q, +3, +5, gain 6p22.1, +9, gain 15q, +19, -4, loss 14q	-
	FISH	No detectable 14q32 translocations	No detectable 14q32 translocations	-
12	-	diagnosis	PD1	-
	SNV	RYR2, CYLD	RYR2, CYLD , ZFHX3	-
	CNV	+2, +3, gain 6p, +8, +9 (partial), +11, gain 15q, +18, +19, -4, loss 6q, -10, loss 13q, loss 16q	gain 6p, gain 15q, +7, +18, +19, -4, loss 6q, -10, -12, loss 1p, loss 1q (partial), loss 13q, loss 14q, loss 16q, -17 (partial), -20, loss 21q	-
	FISH	No detectable 14q32 translocations	No detectable 14q32 translocations	-
13	-	diagnosis	PD1	-
	SNV	KRAS, MPL , GNAS, PABPC1	KRAS , MPL , GNAS , SOX9	-
	CNV	+3, +5, gain 6p (partial), +9, +11, gain 15q, gain 19q (partial), loss 1p, -17	+3, +9, +11, gain 15q, loss 1p, loss 17p (partial)	-
	FISH	No detectable 14q32 translocations	No detectable 14q32 translocations, TP53 deletion	-
14	-	diagnosis	PD1	-
	SNV	BCL7A, SP140, TP53 (c.818G>A), FUBP1 , NTRK2	BCL7A, SP140, ATR , KDM5A , TP53 (c.711G>A), ZNF292	-
	CNV	gain 6p22.1, loss 17p (partial), loss 14q, loss 22q	gain 6p22.1, loss 17p (partial), loss 17q (partial) loss 1p (partial), loss 13q, loss 22q	-
	FISH	No detectable 14q32 translocations, TP53 deletion	No detectable 14q32 translocations, TP53 deletion	-
17		diagnosis	PD3	
	SNV	ALK , KLHL6 , DNAH5	ALK , KLHL6 , DNAH5	-
	CNV	gain 6p, gain 8q (partial), loss 6q, -8 (partial), loss 16q	gain 6p, gain 8q (partial), loss 6q, -8 (partial), loss 16q	-
	FISH	t(11;14)	t(11;14)	-
18	-	diagnosis	PD2	-
	SNV	KRAS	KRAS , PABPC1 (c.1240C>T) , PABPC1 (c.1223A>T)	-
	CNV	gain 6 p (partial)	-	-
	FISH	Translocation 14q32 other than t(4;14) and t(14;16)	Translocation 14q32 other than t(4;14) and t(14;16)	-
19	-	diagnosis	PD3	-
	SNV	CSMD3, EGR1	CSMD3, EGR1, KRAS	-
	CNV	gain 1q, +3, +5, +7, +9, gain 15q, +18, +19, loss 1p (partial), loss 13q	gain 1q, +3, +7, gain 6p, gain 6q (partial) gain 15q, +18, +19, loss 10p, loss 13q	-
	FISH	No detectable 14q32 translocations	No detectable 14q32 translocations	-
20		diagnosis	PD3	
	SNV	EGR1 , LRRK2 , KRAS , DIS3	EGR1, LRRK2, CD79A , PBRM1	-
	CNV	+11, loss 14q (partial)	loss 14q (partial), loss 1 p (partial), loss 2q (partial), loss 6q, loss 10q (partial), loss 12q (partial), loss 13q, -17, gain 6p, gain 11q, gain 15q	-
	FISH	Translocation 14q32 other than t(4;14) and t(14;16)	Translocation 14q32 other than t(4;14) and t(14;16), TP53 deletion	-
21		diagnosis	PD3	-
	SNV	ARID1A (c.2397G>T) , ARID1A (c.2390G>C) , KRAS , MAP3K14, RYR2, TET2 , ABCF1 , ZFHX3 (c.8780G>C) , ZFHX3 (c.2556C>G) , DICER1 , PABPC1	ARID1A (c.2397G>T), ARID1A (c.2390G>C), KRAS , MAP3K14, RYR2 , TET2 , ABCF1 , ZFHX3 (c.8780G>C) , ZFHX3 (c.2556C>G) , DICER1 , ALK , EIF4G2 , NFKB2 , NOTCH3 , KMT2B , KDM5C , ARID1A (c.4208T>G) , DNMT3A , FAT4, PRDM9 , KMT2D , TP53 (c.814G>A) , TP53 (c.550G>C) , RYR1 , RUNX1 , HUWE1 , SOX9 , STAT6 , UBR5	-
	CNV	gain 1q, gain 2p, +3, gain 4p, +5, +6, +7, +9, +11, gain 15q, +19, +21 (partial), loss 1p, loss 2q, loss 4q, -8, -10, -12, loss 13q, loss 14q, -16, -17, -18, -20, loss 22q	gain 1q, +5, loss 1p, loss 4q, -8, -10, -12, loss 13q, loss 14q, loss 16q (partial), -17, -18, -20	-
	FISH	Translocation 14q32 other than t(4;14) and t(14;16)	Translocation 14q32 other than t(4;14) and t(14;16)	

b) Cases with diagnostic sample (a total of 4 samples)

Patient ID	Data type	1st sample	2nd sample	3rd sample	4th sample
16	-	diagnosis	PD1	PD2	PD3
	SNV	BRCA2, DIS3 , RYR2 , ATRX, DNAH11 , SPEN , FAT4, SOX9 , ZFHX3 , RUNX1 , LRP1B , EDC4	BRCA2, DIS3 , RYR2 , ATRX DNAH11	BRCA2, DIS3 , RYR2 , ATRX , DNAH11 , LRP1B , EDC4 , CYLD (c.2065C>T)	BRCA2, DIS3 , RYR2 , ATRX , LRP1B , EDC4 , CYLD (c.2065C>T) , CYLD (c.2465A>G) , FAT4, SOX9 , ZFHX3 , SPEN
	CNV	gain 6p22.1, +9, gain 16p, gain 17q, loss 16q, loss 17p (partial)	gain 6p22.1, +7, +9, gain 17q, loss 16q, loss 17p (partial), loss 6q (partial), loss 18q,	gain 6p22.1, +7, +9, gain 17q, loss 16q, loss 17p (partial), loss 6q (partial)	+7, +9, gain 17q, loss 16q, loss 17p (partial), loss 6q (partial)
	FISH	t(11;14) plus TP53 deletion	t(11;14) plus TP53 deletion	t(11;14) plus TP53 deletion	No data avilable
22	-	diagnosis	PD2	PD3	PD4
	SNV	PABPC1 , BRCA2	PABPC1 , BRCA2 , BRAF , XBP1	PABPC1 , BRCA2 , BRAF , XBP1	PABPC1 , BRCA2 , BRAF , XBP1
	CNV	gain 1q, +3, loss 1p, gain 6p22.1, loss 7p (partial), loss 13q, -18	gain 1q, +3, gain 6p22.1, gain 9q, loss 1p, loss 7p (partial), loss 13q	gain 1q, +3, gain 6p22.1, loss 1p, loss 7p (partial), loss 13q,	gain 1q, +3, gain 6p22.1, loss 1p, loss 7p (partial), loss 13q, loss 14q, -18
	FISH	t(4;14)	No data available	t(4;14)	No data available

c) Cases with only relapsed samples

Patient ID	Data type	1st sample	2nd sample	3rd sample
23	-	PD3	PD4	PD5
	SNV	KRAS, PABPC1 , HIST1H1E	KRAS, PABPC1 , HIST1H1E , SF3B1	KRAS, PABPC1 , HIST1H1E , SF3B1
	CNV	+1, loss 13q, loss 22q (partial)	+1, loss 3p26, loss 13q, loss 22q (partial)	+1, gain 6p22.1, gain 19q14, loss 13q, loss 22q (partial)

	FISH	Translocation 14q32 other than t(4;14) and t(14;16)	Translocation 14q32 other than t(4;14) and t(14;16)	Translocation 14q32 other than t(4;14) and t(14;16)
24	-	PD1	PD2	
	SNV	HIST1H1C, DNAH11 , EGFR, DUSP2 (c.662G>C), CCND1 (c.28G>A), CCND1 (c.131A>C), ATM , DUSP2 (c.720A>G), CYLD, ZFHX3 (c.10523G>C) ,	HIST1H1C , DNAH11 , EGFR, DUSP2 (c.662G>C) , CCND1 (c.28G>A) , CCND1 (c.131A>C) , ATM , DUSP2 (c.720A>G) , CYLD , PAK2	-
	CNV	gain 1q, gain 3q (partial), gain 6p (partial), +7, gain 8q, +18, loss 16q, loss 8p	gain 1q, gain 3q (partial), +7, +18, loss 16q	-
	FISH	Translocation 14q32 other than t(4;14) and t(14;16)	No data available	
25	-	PD1	PD2	
	SNV	FANCA, ATM,	FANCA, ATM, PABPC1	-
	CNV	gain 1q (partial), gain 6p (partial), gain 9p, gain 16p, gain 17q, loss 1p (partial), loss 2q, loss 4q (partial), loss 5q, loss 11q (partial), -12, loss 13q, loss 16q (partial), loss 20p, loss 22q	gain 1q (partial), gain 5p, gain 9p, gain 17q, loss 1p (partial), loss 2q, loss 4q (partial), loss 5q, loss 11q (partial), -12, loss 13q, loss 16q (partial), loss 17p (partial), loss 20p, loss 22q	-
	FISH	t(4;14)	t(4;14) + delTP53	
26	-	PD2	PD3	
	SNV	RET, KRAS , BRIP1, TET2 , TP53 , HIST1H1C, ETV6	RET, KRAS, BRIP1 , TET2 , TP53 , HIST1H1C, ETV6	-
	CNV	+1, +6, +9, gain 15q, +19, loss 13q, loss 14q, loss 17p (partial), loss 18q	gain 1q (partial +6, +9, gain 15q, gain 18p, +19, loss 13q, loss 14q, loss 17p partial,	-
	FISH	No detectable 14q32 translocations, TP53 deletion	No detectable 14q32 translocations, TP53 deletion	
27	-	PD4	PD5	
	SNV	KMT2C, ERN1	KMT2C, ERN1, TP53	-
	CNV	gain 1q, gain 6p22.1, loss 13q, gain 15q, gain 17q (partial), loss 17q (partial), loss 18q, +19	gain 1q (partial), gain 15q, gain 17q (partial), loss 13q, loss 17p, loss 17q (partial), +19	-
	FISH	t(4;14), TP53 deletion	t(4;14), TP53 deletion	
28	-	PD1	PD2	
	SNV	RET, BRCA2, FGFR3	RET, BRCA2, FGFR3	-
	CNV	gain 1q	gain 1q, gain 15q, gain 17q (partial), loss 3p (partial), loss 8p (partial), loss 13q, loss 16q (partial), loss 18q	-
	FISH	t(4;14)	t(4;14)	
29	-	PD5	PD6	
	SNV	SETD2 , TET2 , KMT2C (c.6368C>G), KMT2C (c.5013G>T) , KMT2B , SPEN , ID3, ATR , FAT4, SYNE1, POT1, BRAF, EDC4 , HMCN1 , ATM (c.6745G>T) , HUWE1 , DNAH11 , DICER1 , SOX9 , ZFHX3 (c.8440G>A), ZFHX3 (c.2556C>G) , RBX1, USP9X, HMCN1 , PIK3CA (c.42C>G) , ZNF292 , RYR2 , ABCF1 , ATM (c.6739A>C) , PIK3CA (c.40C>A) , PIK3CA (c.44T>G) , NCOR2	SETD2 , TET2 , KMT2C (c.6368C>G), KMT2C (c.5013G>T), KMT2B , SPEN , ID3, ATR , FAT4, SYNE1, POT1, BRAF, KMT2C, EDC4 , HMCN1 , ATM (c.6745G>T) , HUWE1 , DNAH11 , DICER1 , SOX9 , ZFHX3 (c.8440G>A), ZFHX3 (c.2556C>G) , RBX1, USP9X, HMCN1 , PIK3CA (c.42C>G) , ZNF292 , TP53	-
	CNV	gain 1q, gain 6p22.1, loss 1p (partial), loss 13q, loss 17p (partial)	gain 1q, loss 1p (partial), gain 6p22.1, loss 13q, loss 17p, +18	
	FISH	Translocation 14q32 not specified, TP53 deletion	Translocation 14q32 not specified, TP53 deletion	
30	-	PD5	PD6	PD7

	SNV	LRP1B , HOXA10-HOXA9, NCOR2, FGFR3, PTPRT, EDC4 , PTPRT, ZFHX3 (c.8780G>C) , ZFHX3 (c.2556C>G) , BRAF , PABPC1	LRP1B , HOXA10-HOXA9, NCOR2, FGFR3, PTPRT, EDC4 , PTPRT, ZFHX3 (c.8780G>C) , ZFHX3 (c.2556C>G) , BRAF , PABPC1 , SPEN , LRRK2	LRP1B , HOXA10-HOXA9, NCOR2, FGFR3, PTPRT, EDC4 , PTPRT, ZFHX3 (c.8780G>C) , ZFHX3 (c.2556C>G) , SPEN , LRRK2 , ARID1A (c.3091A>C) , ARID1A (c.3094G>C) , KMT2D (c.9370G>A) , KMT2D (c.5875G>A) , DICER1 , SETD2 , LRRK2 , ARID1A (c.3098A>T) , HACE1 (c.49C>A) , HACE1 (c.40T>G) , HACE1 (c.28C>G) , KMT2A
	CNV	gain 1q, +3, gain 4q, +5, gain 6p, +7, gain 9p, gain 15q, gain 17q (partial), +19, +21 (partial), loss 4p, loss 13q, loss 14q, -18	gain 1q, +3, gain 4q, +5, gain 6p, +7, gain 9p, gain 15q, gain 17q (partial), +19, +21 (partial), loss 4p, loss 13q, loss 14q, -18	gain 1q, gain 6p (partial), gain 16p
	FISH	t(4;14)	No data available	t(4;14)
31		PD4	PD5	PD6
		LRP1B , IRF4 , SYNE1 , FAT4 , CDKN2A , LRRK2 , CYLD ,	LRP1B , IRF4 , SYNE1 , FAT4 , CDKN2A, LRRK2 , CYLD, DNAH11	LRP1B , IRF4 , SYNE1 , FAT4 , CDKN2A , LRRK2 , CYLD , DNAH11
		gain 1q, gain 2p (partial), gain 6p, gain 6q (partial), gain 8q, loss 6q (partial), loss 14q, loss 18q	gain 2p (partial), gain 6p, gain 8q, gain 15q, loss 6q (partial), loss 8p, loss 13q, loss 14q	gain 1q, gain 6p, gain 6q (partial), gain 8q, gain 9q, loss 6q (partial), loss 13q, loss 14q
		No detectable 14q32 translocations	No detectable 14q32 translocations	No data available
32		PD7	PD8	
		PRDM9 , PABPC1 , ARID2 , RYR1, CSMD3	PRDM9 , PABPC1 , ARID2 , RYR1, CSMD3	-
		gain 1q21, +6, +7, gain 15q, +19, loss 13q	+3, +5, +6, +7, +11, gain 15q, +19, loss 13q, loss 22q	-
		No detectable 14q32 translocations	No detectable 14q32 translocations	-
33		PD1	PD3	
		KRAS, ZFHX3 , TCF3 , SHANK2 , LRP1B (c.3075T>G) , MAST4 , DNAH5	KRAS , ZFHX3 , TCF3 , SHANK2 , LRP1B (c.3075T>G) , MAST4 , DNAH5, ATR , RYR2 , EDC4 , LRP1B (c.3058C>A) , SOX9 , ARID1A , NCOR2	-
		+5 (partial), gain 6p22.1, gain 9q, +11, gain 15q, +19, gain 21q, loss 1p, loss 13q	gain 1q21, gain 2p (partial), +5, gain 6p22.1, +9, +11, gain 15q, +19, +21 (partial), loss 2p (partial), loss 17q (partial)	-
		No detectable 14q32 translocations	No detectable 14q32 translocations	

Description: In orange, blue and white are SNVs/CNVs acquired, lost and unchanged over time, respectively. SNVs present in at least two samples with variant allele frequency of at least 40% are underlined. Abbreviations: b, biochemical progression; CNV, copy number variants; FISH; fluorescence in-situ hybridization; PDn, nth disease progression; SNV, single nucleotide variants.
